# A chromosome-level *Mitragyna parvifolia* genome unveils spirooxindole alkaloid diversification and mitraphylline biosynthesis

**DOI:** 10.1093/plcell/koaf207

**Published:** 2025-08-18

**Authors:** Larissa C Laforest, Tuan-Anh M Nguyen, Gabriel Oliveira Matsumoto, Pavithra Ramachandria, Andre Chanderbali, Siva Rama Raju Kanumuri, Abhisheak Sharma, Christopher R McCurdy, Thu-Thuy T Dang, Satya Swathi Nadakuduti

**Affiliations:** Plant Molecular and Cellular Biology Program, Institute of Food and Agricultural Sciences, University of Florida, Gainesville, FL 32611, USA; Department of Chemistry, University of British Columbia, Kelowna, BC V1V 1V7, Canada; Department of Horticultural Sciences, Institute of Food and Agricultural Sciences, University of Florida, Gainesville, FL 32611, USA; Environmental Horticulture Department, Institute of Food and Agricultural Sciences, University of Florida, Gainesville, FL 32611, USA; Research Computing, UF Information Technology, University of Florida, Gainesville, FL 32611, USA; Department of Pharmaceutics, College of Pharmacy, University of Florida, Gainesville, FL 32611, USA; Department of Pharmaceutics, College of Pharmacy, University of Florida, Gainesville, FL 32611, USA; Department of Pharmaceutics, College of Pharmacy, University of Florida, Gainesville, FL 32611, USA; Department of Medicinal Chemistry, College of Pharmacy, University of Florida, Gainesville, FL 32611, USA; Department of Chemistry, University of British Columbia, Kelowna, BC V1V 1V7, Canada; Plant Molecular and Cellular Biology Program, Institute of Food and Agricultural Sciences, University of Florida, Gainesville, FL 32611, USA; Environmental Horticulture Department, Institute of Food and Agricultural Sciences, University of Florida, Gainesville, FL 32611, USA; Genetics Institute, University of Florida, Gainesville, FL 32610, USA

## Abstract

Monoterpene indole alkaloids (MIAs) found in the Rubiaceae have varied pharmaceutical uses. Spirooxindole alkaloids are a structural subtype of MIAs with a unique spiro[pyrrolidine-3,3′-oxindole] ring system. Despite their intriguing structures and potent bioactivities, the evolution and diversification of spirooxindole alkaloids remain poorly understood. We report a high-quality chromosome-scale genome assembly of *Mitragyna parvifolia,* a tree species of the Rubiaceae family that predominantly produces the spirooxindole alkaloid mitraphylline. Comparative genomics, including comprehensive synteny and phylogeny analyses across the MIA-producing order Gentianales revealed a whole-genome duplication event underlying the divergence of the Cinchonoideae alliance from the Coffeeae alliance, leading to diversification of MIA biosynthesis. Transcriptome analyses of young and mature leaves, stems, stipules, and roots integrated with MIA profiling and genome analyses revealed several candidates in the MIA biosynthetic pathway. Functional characterization of selected candidates led to the elucidation of the biosynthesis of the antiproliferative spirooxindole mitraphylline in *M. parvifolia*. These genomic and transcriptomic resources are invaluable to identify the evolutionary origins and diversification of MIAs and spirooxindole alkaloids.

## Teaser

The high-quality genome assembly of *Mitragyna parvifolia* reveals the biosynthetic pathway of mitraphylline, providing insight into the evolution and diversification of monoterpenoid indole alkaloids.

## Introduction

**Figure 1.**
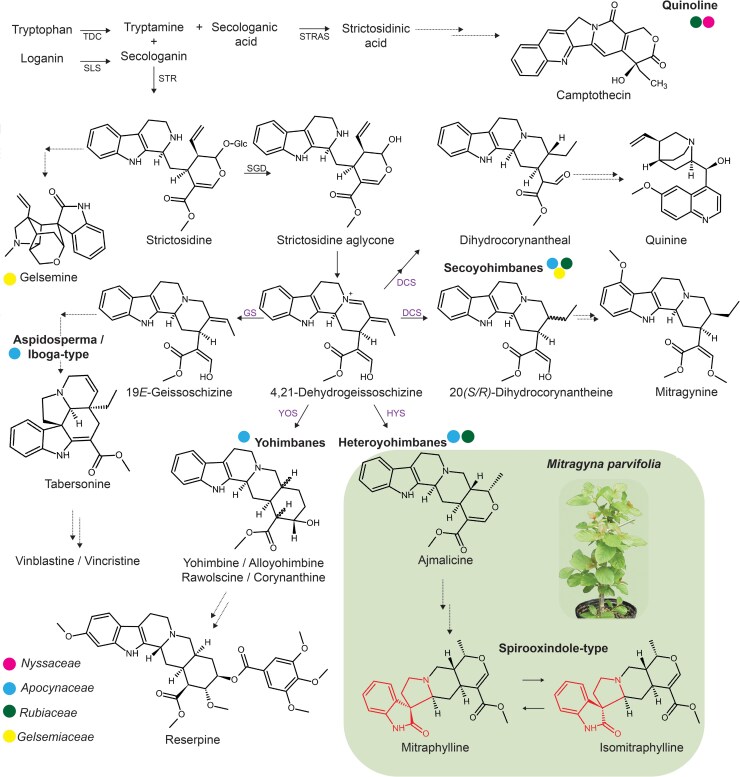
Overview of monoterpene indole alkaloid (MIA) pathway diversification. The indole moiety is derived from tryptamine formed via decarboxylation of tryptophan by enzyme tryptophan decarboxylase (TDC). The monoterpene moiety is derived from secologanin, an end product of the secoiridoid pathway formed by secologanin synthase (SLS). Strictosidine is formed by condensation of secologanin and tryptamine catalyzed by strictosidine synthase (STR). An exception to this is camptothecin biosynthesis in *Camptotheca acuminata* where strictosidinic acid is the central precursor produced by strictosidinic acid synthase (STRAS). Deglycosylation of strictosidine by strictosidine-β-D-glucosidase (SDG) yields strictosidine aglycone which is reduced by medium-chain dehydrogenase/reductase superfamily (MDR) enzymes to form the diverse MIA scaffolds (in bold) that undergo further decoration. MDRs shown are: dihydrocorynantheine synthase (DCS), heteroyohimbine synthase (HYS), yohimbine synthase (YOS), and geissoschizine synthase (GS). Examples of final products are included for each MDR branch and plant families in which they accumulate are indicated. The spiro[pyrrolidin-3,3′-oxindole] moiety is highlighted.

Monoterpene indole alkaloids (MIAs) represent a diverse family of plant specialized metabolites with extensive structural complexity and potent biological activities (O’Connor and Maresh 2006) (Fig. 1). Over 3,000 MIAs are known, and several have been developed into diverse pharmaceuticals (Dudley et al. 2022). For example, vincristine and vinblastine from *Catharanthus roseus* are chemotherapeutic drugs (O’Connor and Maresh 2006), reserpine from *Rauvolfia* species was one of the first plant derived antihypertensive drugs and a first-generation antipsychotic (López-Muñoz et al. 2004), while quinine, from *Cinchona* genus, was the first effective treatment for malaria (Rolleston 1931). Several other MIAs are under investigation for their therapeutic properties. As such, elucidating the biosynthetic pathways that give rise to MIAs has become essential not only for deepening our understanding of the metabolic logic of the host plants, but also for leveraging synthetic biology approaches to produce them more efficiently.

The biosynthesis of MIAs begins with the condensation of tryptamine and secologanin as the first committed step resulting in the formation of strictosidine, the central intermediate for MIAs. Strictosidine deglycosylation yields an unstable aglycone which isomerizes to an unsaturated iminium, 4,21-dehydrogeissoschizine. This compound serves as a divergence point to form distinct MIA scaffolds (Schotte et al. 2023) including corynanthe, aspidosperma, quinoline, iboga, and spirooxindole scaffolds found in plants belonging to Apocynaceae, Rubiaceae, Gelsemiaceae and Nyssaceae (Fig. 1). Unique among these MIAs is the spirooxindole subtype with a characteristic spiro[pyrrolidin-3,3′-oxindole] unit that distinguishes them from the tetrahydro-*β*-carboline or tryptoline moiety observed in other MIA subtypes such as corynanthe and yohimbine alkaloids. Over 100 spirooxindole alkaloids with a wide range of bioactivities have been reported from various plant genera including *Mitragyna*, *Rauvolfia*, *Uncaria,* and *Vinca* since the first spirooxindole alkaloid discovery from the root of yellow jessamine (*Gelsemium sempervirens*) in 1870 (Bindra 1973). This scaffold (Fig. 1) has emerged as a versatile pharmacophore in drug development (Ding et al. 2006; Gollner et al. 2016; Hati et al. 2016; Kelemen et al. 2018). Examples include the anti-Alzheimer's rhynchophylline (Fu et al. 2014), the antihypertensive and antidepressant isorhynchophylline (Xian et al. 2017), and the antiproliferative and antiamyloidogenic mitraphylline and isomitraphylline (García Prado et al. 2007; Xian et al. 2017; Li et al. 2020). *Gelsemium*-derived spirooxindole alkaloids such as gelsemine and koumine, feature a fused oxindole–spirocyclic framework with a characteristic bridged or cage-like structure, contributing to their neurotoxic properties (Chen and Hong 2022). In contrast, Rubiaceae-derived spirooxindole alkaloids, such as mitraphylline and pteropodine often feature a simpler spirocyclic oxindole system (Zhou et al. 2020) (an oxindole ring sharing one carbon atom at the C_3_ position with a cycloalkyl group or a heterocycle) specific for this family.

*Mitragyna* species, including *Mitragyna speciosa, Mitragyna parvifolia, Mitragyna diversifolia, Mitragyna hirsuta,* and *Mitragyna rotundifolia* naturally produce various MIAs specific for Rubiaceae family (Brown et al. 2017). The wide range of MIA and spirooxindole scaffolds occurring across the *Mitragyna* genus is an important ethnobotanical source for potential drug discovery in addition to providing an excellent system to understand the evolution and diversification of these pharmaceutically relevant MIAs. *M. parvifolia*, a tree species endemic to Indian subcontinent is known to produce spirooxindole alkaloids predominantly, mitraphylline, a compound known for its antiproliferative effects and *in vivo* efficacy to induce apoptosis in human breast cancer, sarcoma, and leukemia cell lines (García Prado et al. 2007). Mitraphylline is hypothesized to be formed by oxidative rearrangement of the tetrahydro-*β*-carboline moiety of ajmalicine (Lopes et al. 2019; Nguyen et al. 2023), which is formed via the reduction of 4,21-dehydrogeissoschizine by heteroyohimbine synthase (HYS) or tetrahydroalstonine synthase (THAS) (Stavrinides et al. 2016) (Fig. 1). However, the final steps leading to the formation of mitraphylline remain unknown. It is also unclear why *M. parvifolia* has a unique mitraphylline-rich chemical profile as compared to other species in the same family and what makes spirooxindoles in Rubiaceae unique from others.

In this study, we sequenced and assembled a high-quality chromosome-scale genome of *M. parvifolia,* which predominantly accumulates spirooxindole alkaloid mitraphylline. We constructed 22 pseudochromosomes (2*n* = 4*x* = 44) using high-throughput chromosome conformation capture (Hi-C). Using this reference genome, we performed comparative genomics analyses, including comprehensive synteny and phylogeny across the Gentianales to probe the role of genome structure in the diversification of the poststrictosidine pathways in MIA biosynthesis. We identified species divergence of the Cinchonoideae alliance from the Coffeeae alliance by whole-genome duplication (WGD) event. We then generated RNA-seq and corresponding targeted metabolite datasets from various MIA accumulating tissues of *M. parvifolia* and performed differential gene expression and correlation analyses to investigate the mitraphylline biosynthetic pathway. We elucidated the final step(s) of the biosynthesis of the antiproliferative spirooxindole alkaloid mitraphylline and its isomers, emphasizing the importance of these genomics and metabolite data sets for pathway discovery of diverse pharmaceutical MIAs in Rubiaceae.

## Results

### Chromosome-scale genome assembly of *M. parvifolia*

**Figure 2.**
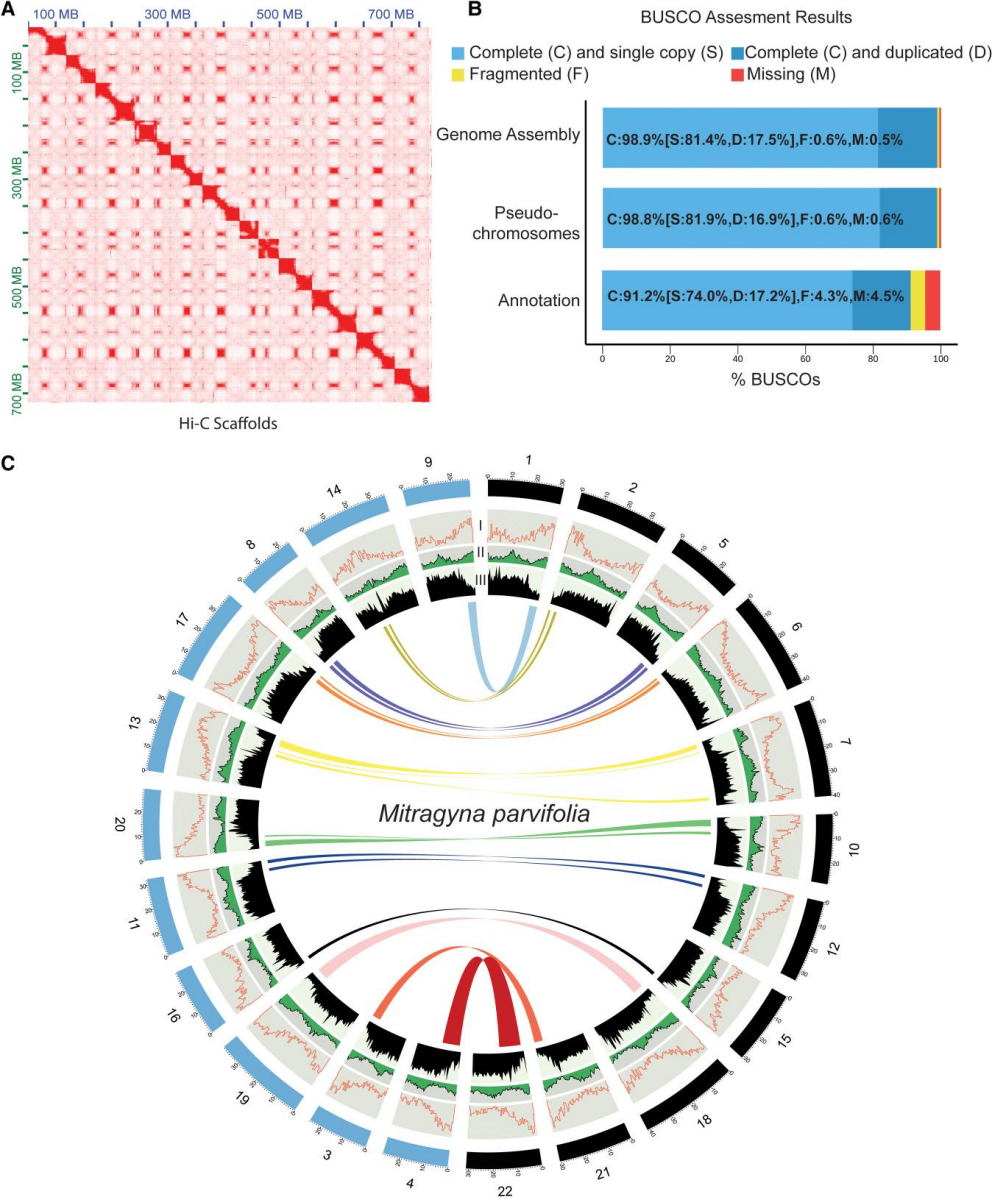
Composition, synteny and distribution of genomic and epigenomics features of the *Mitragyna parvifolia* genome. **A)** Scaffolding assembly Hi-C contact map generated from mature leaves of *M. parvifolia* genome. **B)** Percentage of 1,614 embryophyta BUSCO (Benchmarked Universal Single Copy Orthologs) in the final scaffolded genome assembly, pseudochromosomes (scaffolds larger than 5 Mb), and genome annotation. **C)** Circular representation of 22 pseudochromosomes of the *M. parvifolia* genome and colored lines indicate syntenic blocks representing groups of collinear orthologs within the *M. parvifolia* genome. Circular tracks indicate (from outer to inner) I) Gene count per 500 Kb (orange), II) GC content per 500 kb (green), III) Proportion of transposable elements density (black).

We generated Oxford Nanopore Technologies (ONT) long-reads (∼100×) and Illumina short reads (∼15×, Supplementary Table S1) to construct a *de novo* genome assembly for *M. parvifolia.* Following error correction and haplotig purging, we produced a draft assembly consisting of 10,164 contigs totaling 774.1 Mb (Supplementary Table S1), with a contig N50 length of 181 Kb (maximum length = 4.94 Mb) (Table 1). The *M. parvifolia* genome is predicted to be an allotetraploid with AABB genome structure (AABB = 11.9%; AAAB = 2.25%) with a predicted genome size of ∼1.02 Gb using *K*-mer spectral analyses (Supplementary Fig. S1A and B). Our assembly size (2*x* = 774.1 Mb) was slightly higher than the estimated genome size using *K*-mer analysis (2*x* = 510 Mb), potentially due to retained haplotigs, reflected in the high number of duplicated Benchmarked Universal Single Copy Orthologs (BUSCO) genes (Fig. 2, Supplementary Table S1). *K*-mer based size predictions could also be underestimating genome size due to high repetitive content. However, this assembly size is relatively close to the genome size of *Mitragyna speciosa* (720 to 780 Mb) (Brose et al. 2021; Pootakham et al. 2022).

**Table 1.** Statistics for *Mitragyna parvifolia* genome assembly

Assembly statistic	Nanopore + Illumina	Nanopore + Illumina + Hi-C
Contigs/Scaffolds	10,164	2,557
Assembly size (bp)	774 171 054	778 737 054
N50 (bp)	181 509	31 324 338
L50	954	11
L90	6,204	22
Longest contig/scaffold (bp)	4 941 770	41 328 477
GC content (%)	34.65	34.65

Hi-C (210×; Supplementary Table S1) was used to order and orient the contigs resulting in 22 pseudochromosomes (Fig. 2A, Supplementary Fig. S2), with an N50 of 31.3 Mb, a 172× increase in N50 without the Hi-C (Table 1). 92.6% of the assembly was placed in pseudochromosomes, with a scaffold N50 of 32.7 Mb (maximum length = 41.3 Mb), and there were 2,535 unplaced scaffolds.

In the final assembly, 98.9% of complete BUSCO genes were present on the 22 pseudochromosomes (Fig. 2B), with 17% being duplicated, corresponding to the proposed tetraploid structure of the genome (2*n* = 4*x* = 44) as evidenced in *M. speciosa* (Brose et al. 2021; Pootakham et al. 2022). In addition, the *M. parvifolia* genome assembly was collinear with the recently published chromosome-level genome assemblies of *M. speciosa* (Pootakham et al. 2022) and *Uncaria rhynchophylla* (Hu et al. 2024), a closely related spirooxindole alkaloid-producing species (Supplementary Fig. S1, C and D). These data altogether demonstrated that we have thereby constructed a high quality, contiguous, and complete *M. parvifolia* genome at the chromosomal level (Fig. 2, Supplementary Figs. S1 and S2, Table 1, Supplementary Table S1).

### Repeat annotation and gene annotation

Annotated repetitive content and transposable elements (TEs) revealed approximately 50% of the genome assembly is composed of repetitive sequences (Supplementary Table S2). This high repetitive content in the *M. parvifolia* genome is consistent with genomes of *M. speciosa* (51.21%) (Pootakham et al. 2022), *Coffea eugenioides* (59.7%) (Salojärvi et al. 2024), and *U. rhynchophylla* (56.34%) (Hu et al. 2024), with Gypsy long-terminal repeats making up a significant proportion of all TEs (Supplementary Table S2).

To annotate the *M. parvifolia* genome, we assembled a *de novo* transcriptome from RNA-seq data sets from young and mature leaves, stipules, stems, and roots, which was near complete based on BUSCO evaluation (C: 99.6%, S: 9.9%, D: 89.7%, F: 0.2%; Supplementary Table S1) with high duplication rate likely due to the presence of isoforms. Structural gene annotation resulted in a total of 50,187 gene models comprising 91.2% complete [S: 74.0%, D: 17.2%, F: 4.3%, M: 4.5%] BUSCO genes (Fig. 2B). Roughly 70% of genes were functionally annotated by eggnog-mapper.

TE density was inversely correlated with gene count and gene-rich regions, potentially corresponding to centromeric and pericentromeric regions (Fig. 2C). This correlation is consistent with pericentric regions in closely related genus *Coffea* (Presting 2018; Salojärvi et al. 2024; Scalabrin et al. 2024). Synteny analysis revealed duplicated syntenic blocks throughout the genome with one-to-one synteny between all 22 chromosomes (Fig. 2C) supporting *K*-mer based findings that the *M. parvifolia* genome is highly heterozygous and likely underwent a polyploidization event as predicted by SmudgePlot (Supplementary Fig. S1).

### Phylogenomic analyses and evolution of gene families

**Figure 3.**
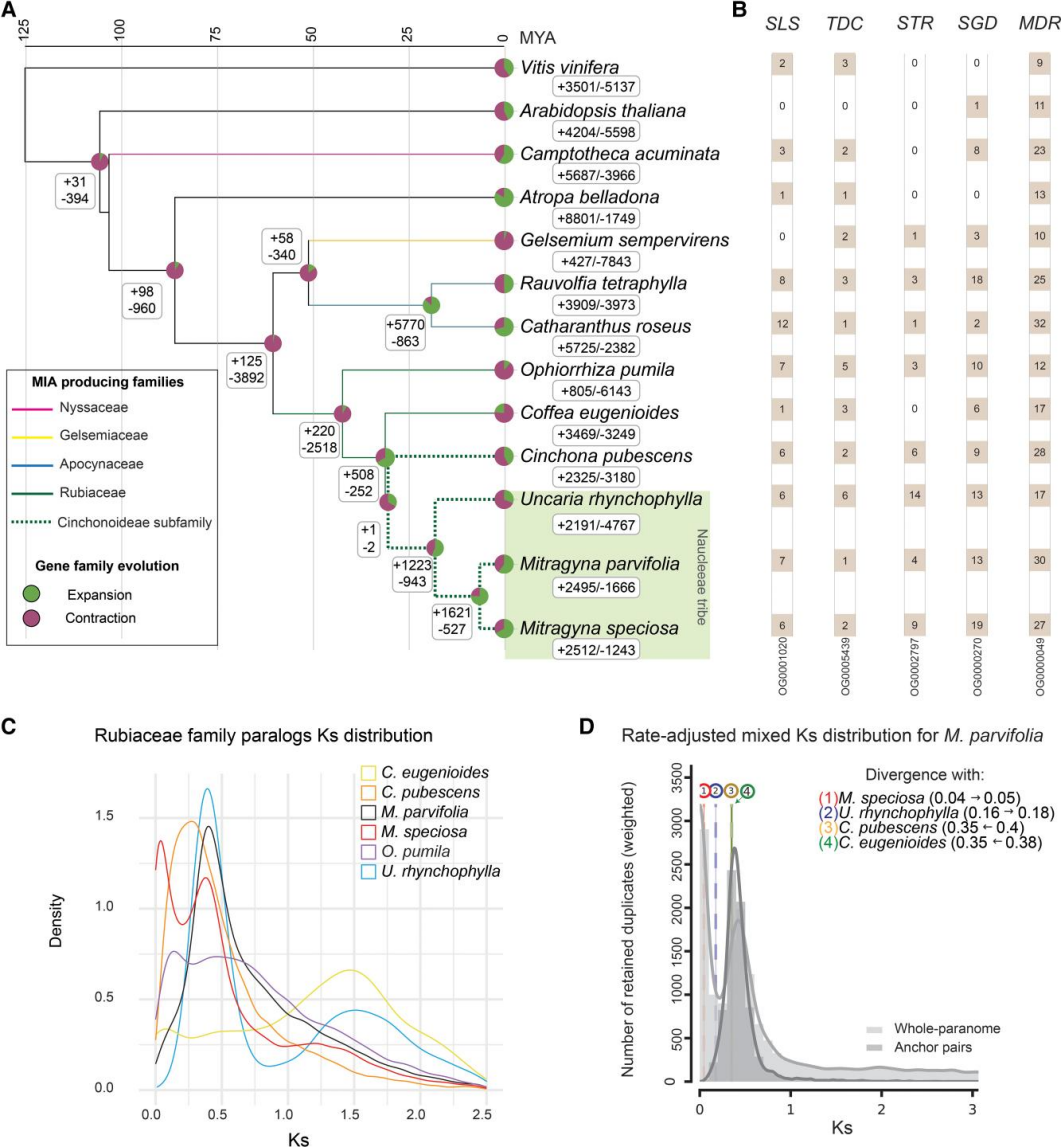
Comparative genomics analyses of *M. parvifolia* with 12 other monoterpene indole alkaloid (MIA) and non-MIA-producing plant species. **A)** Phylogenetic species tree inferred from shared orthologs. Time is depicted as million years ago (MYA). Colored branches represent MIA-producing species belonging to the Cornales order (pink, *C. accuminata*) and the Gentianales families, Gelsemiaceae (yellow, *G. sempervirens)*, Apocynaceae (light blue, *C. roseus* and *R. tetraphylla)*, and Rubiaceae (dark green, *O. pumila, C. pubescens, U. rhynchophylla, M. speciosa*, and *M. parvifolia*). Subfamily taxonomical descriptions are indicated in dashed lines and a colored box. Gene family expansions (green) and contractions (purple) are shown for each lineage. Changes in gene family sizes of ancestral states of orthogroups are depicted at each node. **B)** Number of genes per species for each putative MIA-gene family present in *M. parvifolia*. For genes with multiple annotated orthogroups, the family containing the top homologous *M. parvifolia* genes are illustrated. SLS, Secologanin synthase; TDC, tryptophan decarboxylase; STR, strictosidine synthase; SGD, strictosidine-β-D-glucosidase; MDR, medium-chain dehydrogenase/reductase. **C)** Distribution of synonymous substitutions (Ks) between paralogs within each species. *K_s_* distribution revealed a shared polyploidization event in the Naucleeae subtribe, as well as *Cinchona pubescens*. **D)** Estimated divergence based on adjusted *K_s_* distribution of orthologs between *M. parvifolia* and denoted species.

To evaluate the evolution of the *M. parvifolia* genome and gain insights into the drivers of MIA pathway diversification, we performed comparative genomics analyses across the order Gentianales including MIA-producing species within and outside the Naucleeae tribe (Supplementary Table S3). We also included *Camptotheca acuminata*, an MIA-producing plant belonging to Cornales order, in addition to several non-MIA-producing outgroups, including an ancient diploid and purine alkaloid (caffeine) producer *C. eugenioides*, a tropane alkaloid producer *Atropa belladonna* (Solanaceae), and non-alkaloid producers *Arabidopsis thaliana* (Brassicaceae), and *Vitis vinifera* to investigate the evolutionary history of the MIA biosynthetic pathway, and better delineate lineage-specific MIA pathway gene families from those involved in broader alkaloid metabolism (Supplementary Table S3). We clustered the annotated protein coding genes of all the above-mentioned species into a total of 37,163 gene families (orthogroups), with 592,729 (94.2%) genes being assigned to orthogroups and an average of 16 genes per family. A total of 6,053 orthogroups were commonly present in all species. These were then used to infer a phylogenetic tree to evaluate the taxonomic position of *M. parvifolia* in the Naucleeae tribe, as well as to probe the evolution of *M. parvifolia* genome (Fig. 3A) and specifically genes involved in MIA biosynthesis (Fig. 3B).

Initially, a species tree was inferred using the single copy orthologs shared between all species evaluated with IqTree (Supplementary Fig. S3). However, the resulting tree topology was incongruent with the well-established taxonomic relationships in the Rubiaceae family, with *Coffea pubescens* sister to *C. eugenioides* (Supplementary Fig. S3) rather than the Naucleeae clade (Bremer et al. 1995; Razafimandimbison and Bremer 2001; Razafimandimbison et al. 2004; Bremer and Eriksson 2009). Interestingly, the OrthoFinder tree, which is a species tree inferred from all orthogroups with all species present, revealed similarly errant topology, showing *C. eugenioides* as sister to the Naucleeae tribe, and not *C. pubescens* (Supplementary Fig. S4). As the taxonomic relationships between the *Coffea*, *Cinchona*, and *Mitragyna* genera are well established, we inferred a species tree from all shared orthogroups using ASTRAL-Pro3, constrained by the established species tree describing all members other than *M. parvifolia* (Fig. 3A). Although the Naucleeae tribe was monophyletic, our results indicated very poor resolution of the topology of the Cinchonoideae alliance, with no bootstrap support for the placement of *C. pubescens* sister to the Naucleeae tribe. The complexity in resolving this relationship limits our understanding of the evolution of alkaloid metabolism between the quinoline alkaloid-producing *Cinchona* species and the spirooxindole alkaloid-producing Naucleeae tribe.

### WGD and species divergence

WGD events and polyploidization are a major driver of evolution and diversification in plants, and an important mechanism for the diversification of secondary metabolites. As such, we evaluated synonymous substitutions rates (Ks) of paralogous gene pairs in all species (Supplementary Fig. S5). *K_s_* distributions show a shared peak between all species roughly between 1.1 and 1.5 (Supplementary Fig. S5), corresponding to the gamma triplication event shared by eudicots (Jiao et al. 2012).

Interestingly, the members of the Naucleeae tribe shared a more recent peak at ∼0.4 which is absent from the *K_s_* distributions of paralogous genes in the closely related *C. eugenioides* (Fig. 3C, Supplementary Fig. S5). In addition, ∼50% (23,010) of paralogous gene pairs in *M. parvifolia* were predicted to be derived from whole genome or segmental duplications, supporting our inference that a polyploidization event has significantly affected *M. parvifolia* genome architecture/organization (Supplementary Fig. S6). Importantly, a *K_s_* peak is present at 0.4 in *C. pubescens* paralog *K_s_* indicating that *C. pubescens* genome underwent a polyploidization/WGD around the same time as the Naucleeae tribe and after its divergence from their last common ancestor with *C. eugenioides* (Fig. 3C, Supplementary Fig. S5).

To understand the evolutionary relationships within the Cinchonoideae subfamily and estimate the divergence between its member species, we evaluated the *K_s_* of orthologous gene pairs (Fig. 3D). Results indicate that the divergence of the *Mitragyna* genus from the last common ancestor with *U. rhynchophylla* (*K_s_* ≈ 0.18) occurred more recently than that of the divergence of the Naucleeae tribe from the last common ancestor with *C. pubescens* (*K_s_* ≈ 0.35; Fig. 3D), supporting the placement of the tribe sister to *C. pubescens*. Interestingly, however, the divergence of *M. parvifolia* from *C. pubescens* appears to have occurred at roughly the same time as that of *M. parvifolia* from *C. eugenioides* (*K_s_* ≈ 0.35; Fig. 3D; Supplementary Table S4). *K_s_* distributions indicate this result is true for the whole Naucleeae tribe (Supplementary Table S4). The time of divergence estimated by CAFÉ (∼30 MYA, Fig. 3A) corresponds well to the crown age (node age/age of radiation) of the Cinchonoideae alliance (38.7 MYA) (Bremer and Eriksson 2009; Wikström et al. 2015). Thus, we inferred that the Naucleeae tribe diverged from *C. pubescens* soon after its divergence from *C. eugenioides*, reflecting the polytomy at the base of this clade which remains to be resolved.

Lastly, gene depth analysis revealed that *C. eugenioides* had a 1:2 gene to gene ratio with all species of the Cinchonoideae subfamily (Supplementary Fig. S7). Thus, we inferred that one or several independent WGD or polyploidization events may play a major role in the divergence of the Cinchonoideae alliance from the Coffeeae alliance, and the diversification of its members. These results, taken together with ortholog *K_s_* distributions in the Rubiaceae family suggest that polyploidization events in Cinchonoideae were roughly contemporaneous with (or slightly predate) the divergence of these clades. As such, the evolutionary history of these species may be impacted by large differences in rates of evolution, especially in new paralogs stemming from a shared polyploidization/WGD event or may be complicated by 2 independent but contemporaneous polyploidization events.

### Gene family evolution and GO enrichment analysis

The expansion and contraction of specific gene families as genomes evolve contribute to the phenotypic diversity in plants (Panchy et al. 2016). We investigated gene family size changes across our species tree, revealing that 2,495 *M. parvifolia* gene families (containing 10,440 genes) have expanded while 1,666 *M. parvifolia* gene families (containing 2,091 genes) underwent contractions (Fig. 3A). *M. parvifolia* had the second largest number of unique orthogroups of all Rubiaceae species (1,117 comprised of 3,219 genes), followed by *M. speciosa* (Supplementary Fig. S8). We performed gene ontology (GO) enrichment analysis with an *M. parvifolia* specific GO database and found 51 biological processes and molecular functions enriched in *M. parvifolia* unique orthogroups, the most significant of which involve retrotransposition and DNA synthesis, specialized metabolism, and sulfur metabolism (Supplementary Table S5).

A total of 210 biological processes, 82 molecular functions, and 29 cellular components were significantly enriched in expanded *M. parvifolia* orthogroups (Supplementary Table S6). Biological processes involved in developmental regulation (GO:0080186, GO:0010083, GO:0010448, GO:1905613; *P-value* = 7.25e−9), and response to stress from fungal or viral origins (GO:0002239 *P-value* = 8.02e−6; GO:0002230 *P-value* = 9.17e−6; GO:0050691 *P-value* 1.69e−5) were heavily represented in the top 25 significantly enriched biological processes in expanded *M. parvifolia* orthogroups (Supplementary Fig. S9, Supplementary Table S6). Interestingly, within the orthogroups unique to *Mitragyna* species, the most significantly enriched biological processes involved transport of cell wall components (GO:0015752, GO:0015753, GO:0015757, GO:0015795, GO:0015797; *P-value* = 3.35e−8), and defense against virus and fungi (GO:0009615, *P-value* = 4.35e−8; GO:0002239, GO:0002230, *P-value* = 0.0001; GO:0002238, *P-value* = 0.0002) (Supplementary Table S7). Looking at the ancestral state of the Naucleeae tribe (expansions shared between the *Mitragyna* and *Uncaria* genera), most significantly enriched processes in expanded orthogroups were involved in secondary metabolism, response to pathogens, and development (Supplementary Fig. S9, Supplementary Table S8).

### Synteny and MIA-gene family evolution

*M. parvifolia* was previously reported to accumulate several spirooxindole-type alkaloids (Pandey et al. 2006). To analyze MIA biosynthetic gene family evolution and diversification, we identified putative MIA biosynthesis genes in *M. parvifolia* by exploring the top 5 blast hits (%id > 60%) for the characterized biosynthetic genes in the well-studied, MIA-rich *C. roseus* or the closely related *M. speciosa*. MIA biosynthetic genes were distributed across 11 *M. parvifolia* pseudochromosomes. Several of these genes are clustered closely, for instance, the *MATE-STR-SLS* cluster on chromosome 4 or *MATE-STR-TDC* on chromosome 22 (Supplementary Fig. S10).

**Figure 4.**
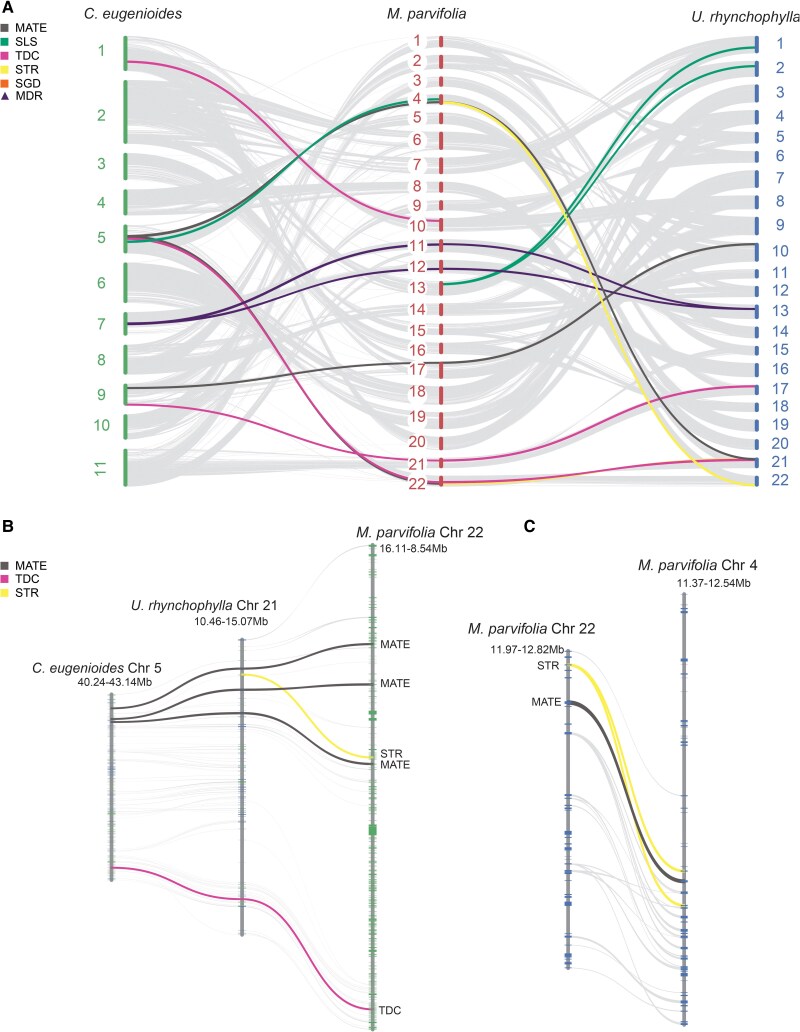
Collinear relationships between selected Rubiaceae genomes. **A)** Macrosyntenic relationships between *Mitragyna parvifolia, Coffea eugenioides* and *Uncaria rhynchophylla* with syntenic blocks containing monoterpene indole alkaloid (MIA) pathway genes highlighted. **B)** Microsyntenic view of *TDC -STR-MATE* cluster, which is conserved between *M. parvifolia, U. rhynchophylla* and *C. eugenioides*. Interestingly, no STR was recovered in syntenic blocks with *C. eugenioides*. **C)** Microsyntenic view of *TDC-STR-MATE* cluster within the *Mitragyna* genome in homoeologous chromosomes. TDC, tryptophan decarboxylase; STR, strictosidine synthase; MATE, multidrug and toxic compound extrusion transporter; SLS, Secologanin synthase; SGD, strictosidine-β-D-glucosidase; MDR; medium-chain dehydrogenase/reductase.

We conducted a three-way synteny analysis between the spirooxindole alkaloids-producing *M. parvifolia, U. rhynchophylla,* and the closely related, purine alkaloid-producing *C. eugenioides*. Our analysis revealed that all core MIA biosynthetic machinery had at least one collinear syntenic region between the 3 species, with the notable exception of *STR* and *SGD*, which were not syntenic between *M. parvifolia* and *C. eugenioides* (Fig. 4A and B). Additional synteny analysis in Rubiaceae including *O. pumila* and *M. speciosa* showed clear markers of duplication of MIA biosynthetic genes between *O. pumila* and the *Mitragyna* species (Supplementary Fig. S11, A and B). Orthologs of the *M. speciosa* gene 3eCIS (3-*epi*-corynoxeine/isocorynoxeine synthase) (Nguyen et al. 2023), a CYP450 responsible for generating the oxindole scaffold from secoyohimbanes, were only syntenic between *M. parvifolia* and *M. speciosa* (Supplementary Fig. S11B), although homologs were present in *U. rhynchophylla* and *C. pubescens*.

The well-known *TDC-STR-MATE* biosynthetic cluster was present, syntenic, and collinear in *U. rhynchophylla* and *M. parvifolia* (Fig. 4B). Interestingly, *STR* and *MATE* were present in duplicate in a collinear block within the *M. parvifolia* genome, indicating that portions of chromosome 4 and 22 share identity by descent and are therefore homoeologous (Fig. 4C). Interestingly, *MpTDC* was the only MIA-gene family member that showed signature of contraction in *M. parvifolia* (Fig. 3B). Gene family sizes for the upstream biosynthetic genes *MpSTR and MpSGD* remained unchanged, congruent with their conserved roles in MIA metabolism. Interestingly, we observed notable expansion of the medium-chain reductases (*MDR*)/Alcohol dehydrogenases (*ADH*) in several of the analyzed species, including the *Mitragyna species* (Fig. 3B, Supplementary Fig. S12). These *MDR*s were predicted on a few scaffolds alongside other MIA biosynthetic genes, including noticeable clusters on pseudochromosome 12 with 7 *MDR*s in tandem, and scaffold 18 with 2 putative *MDR*s downstream of *SGD* (Supplementary Fig. S10). These MDRs include homologs of previously reported MDRs in MIA pathway such as *CrTHAS, CrHYS, MsDCS1*, *CpDCS, RtYOH* (Stavrinides et al. 2016; Trenti et al. 2021; Schotte et al. 2023; Stander et al. 2023).

All identified putative *MDR* genes belonged to a single gene family involved in generating the diverse corynanthe scaffolds via the reduction of the reactive intermediate 4,21-dehydrogeissoschizine. CAFE (Computational Analysis of gene Family Evolution) results (Supplementary Fig. S12) indicated extensive gene family expansion, likely driven by duplication and neofunctionalization, contributing to MIA scaffold diversity. A more in-depth investigation of *MDR/ADH* gene family tree using the branch-site unrestricted statistical test for episodic diversification (BUSTED-MH) and adaptive Branch-Site Random Effects Likelihood (ABSREL) framework revealed significant evidence (*P* < 0.05) of episodic diversifying selection on 31 branches in the *MDR/ADH* gene family, including across several branches of a Gentianales specific clade (Supplementary Fig. S13).

### MIA accumulation and differential gene expression in *M. parvifolia*

**Figure 5.**
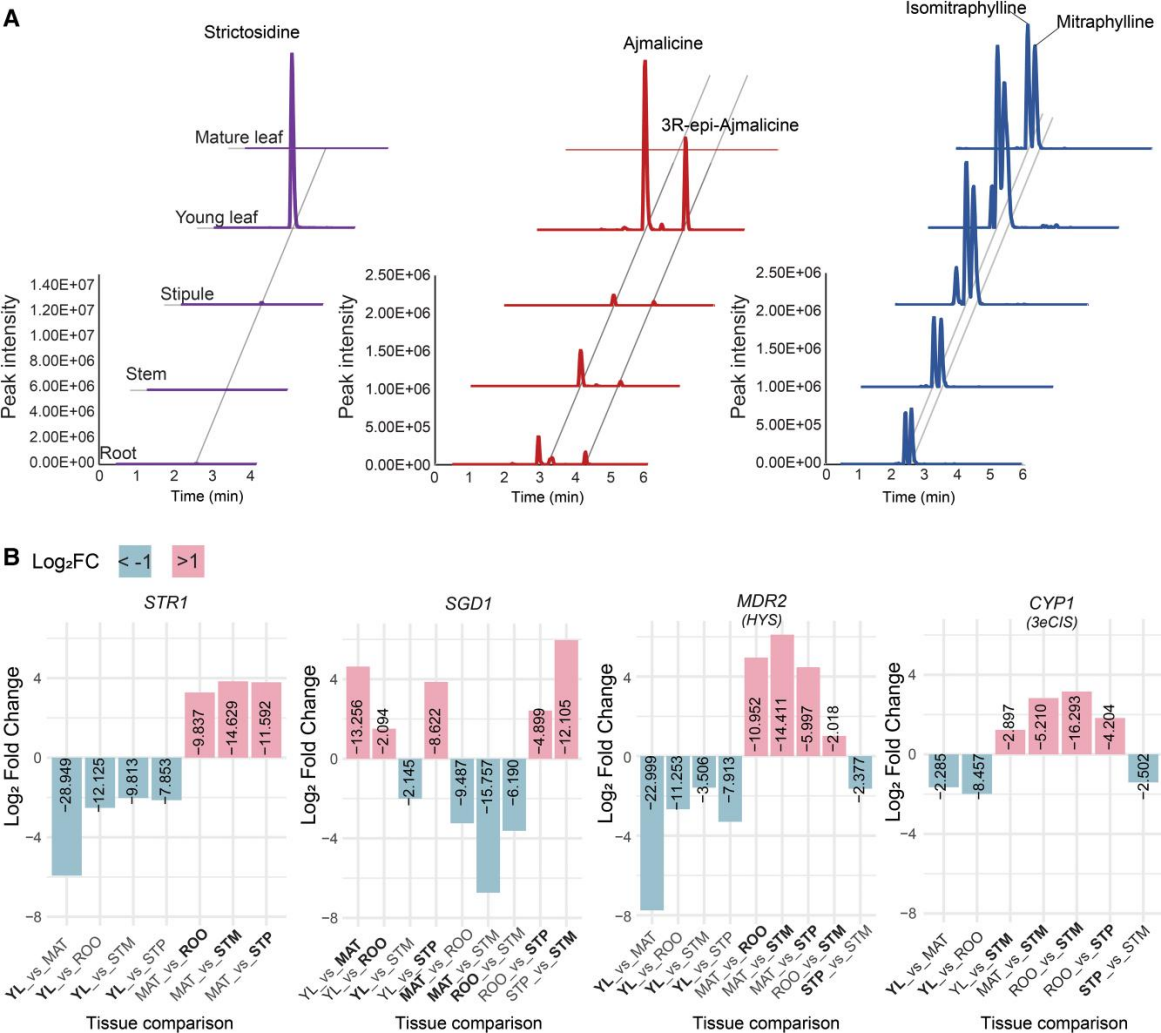
Targeted monoterpene indole alkaloid (MIA) accumulation and differential gene expression (DGE) in various *Mitragyna parvifolia* tissues. **A)** Accumulation of strictosidine, (3*S*/*R*)-ajmalicine, and mitraphylline/isomitraphylline in *M. parvifolia* tissues. **B)** DGE patterns of putative MIA biosynthetic genes. Any comparison in which genes were differentially expressed are shown and log10 *P*-value is shown in the bars. Control tissue appears first in each comparison and tissue with preferential expression is denoted in bold. YL, young leaves; MAT, mature leaves; ROO, roots; STM, stems; STP, stipules.

A targeted set of 17 monoterpene indole alkaloids (MIAs) including the pharmaceutically important alkaloids mitragynine and mitraphylline were quantified from various tissues, including young and mature leaves, roots, stems and stipules (Fig. 5A, Supplementary Table S9). Strictosidine (*m/z* 531) was only detected in young leaves. Similarly, (3*S*)-ajmalicine (*m/z* 353) and its isomer (3*R*)-*epi*-ajmalicine (*m/z* 353), mitraphylline (*m/z* 369) and its isomer isomitraphylline (*m/z* 369) were also highly accumulated in young leaves, suggesting a pathway from strictosidine to mitraphylline via (3*S*)-ajmalicine or its epimer in this tissue (Fig. 5A, Supplementary Table S9). To the best of our knowledge, *M. parvifolia* is a unique *Mitragyna* species with a high spirooxindole mitraphylline chemical profile (Supplementary Table S9).

To investigate the expression patterns of the well understood MIA biosynthetic machinery, from the formation of the central precursor strictosidine to the commitment of the diversifiable metabolite, dehydrogeissoschizine, to a heteroyohimbine fate, we performed a differential gene expression (DGE) analysis. Putative genes *STR*, and *MDR2*, which had highest homology to *MsHYS*, were upregulated in young leaves when compared to all other tissues, with the most significant difference in expression being between young and mature leaves having log_2_FC = −5.93 (log_10_*P-*value = −28.9) and log_2_FC = −7.75 (log_10_*P-*value = −23), respectively. On the other hand, *SGD* was downregulated in young leaves compared to all tissues other than stems (log_2_FC = −2.01 log_10_*P* = −2.14). This corresponds well with previous reports of *SGD* downregulation during elicitation of terpene indole alkaloid (TIA) (Peebles et al. 2009) and MIA (Sun and Peebles 2016) biosynthesis shown in the model MIA species *C. roseus* to be due to *SGD*'s self-inhibition via the expression of a splice-variant (Carqueijeiro et al. 2021). The expression pattern of CYP1, homologous to *Ms3eCIS*, was similar to upstream pathway genes, being upregulated in young leaves, stems, and stipules (Fig. 5).

### The biosynthesis of mitraphylline and its isomer in *M. parvifolia*

**Figure 6.**
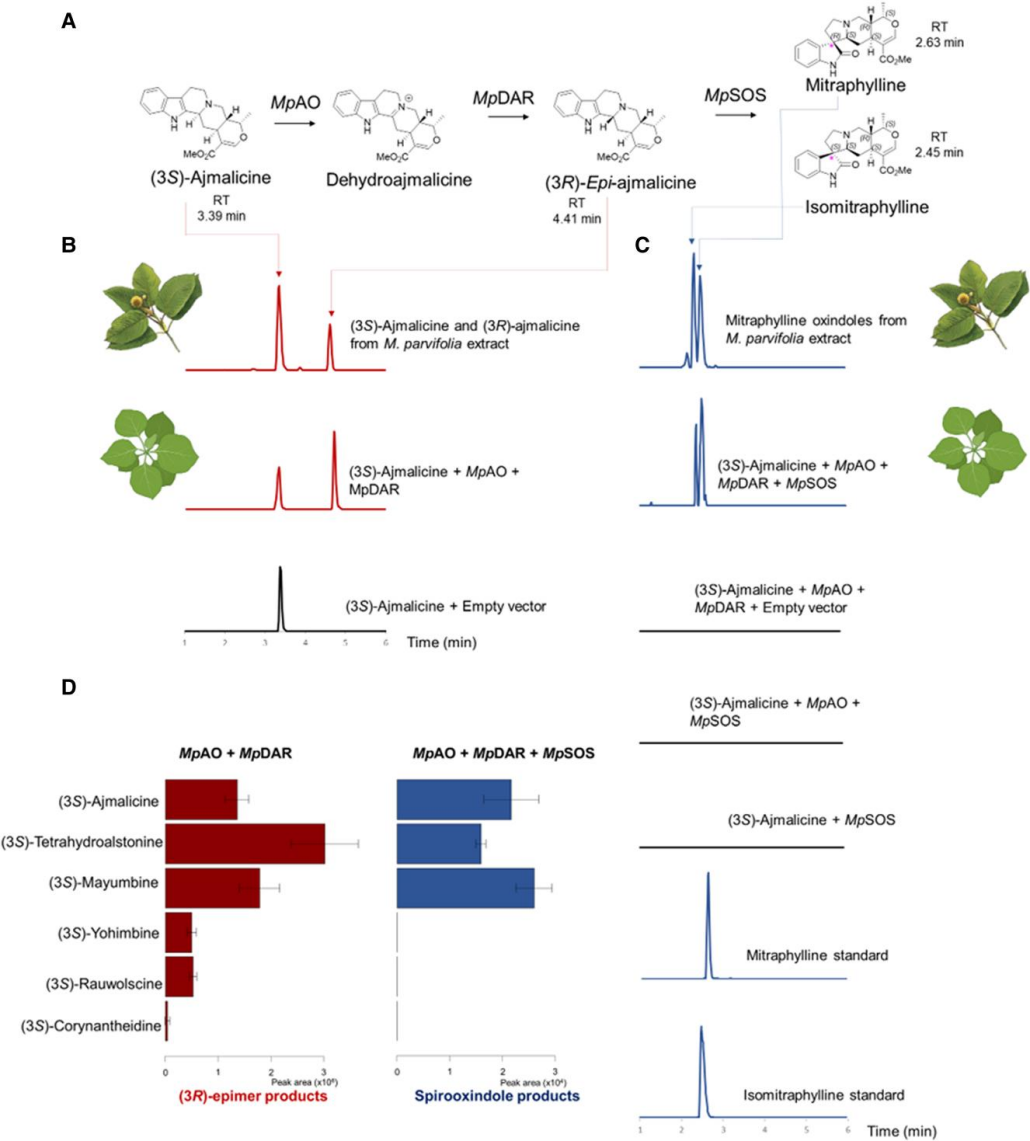
Biosynthesis of mitraphylline in *M. parvifolia*. **A)** Biosynthetic pathway of mitraphylline and isomitraphylline from (3*S*)-ajmalicine involves *Mp*AO (ajmalicine oxidase), *Mp*DAR (dehydroajmalicine reductase), and *Mp*SOS (spirooxindole synthase) enzymes from *M. parvifolia.*  **B)** Extracted ion chromatograms (EIC) for *m/z* [M + H]^+^ 353 showing in vivo activity of *M*pAO and *Mp*DAR on (3*S*)-ajmalicine (Retention time (RT) 3.39 min), producing (3*R*)-*epi*-ajmalicine (RT 4.41 min. **C)** EIC for *m/z* [M + H]^+^ 369 showing the in vivo assays in *N. benthamiana* of *Mp*AO, *Mp*DAR, and *Mp*SOS with (3*S*)-ajmalicine producing pair of spirooxindole alkaloids mitraphylline (RT 2.63 min) and its isomer isomitraphylline (RT 2.45 min). The combination of *Mp*AO, *Mp*SOS or only *Mp*SOS cannot convert the (3*S*)-ajmalicine to the spirooxindoles mitraphylline and isomitraphylline **D)**  *Mp*AO and *Mp*DAR can convert various (3*S*) isomers such as (3*S*)-ajmalicine, (3*S*)-tetrahydroalstonine, (3*S*)-mayumbine, (3*S*)-yohimbine, (3*S*)-rauwolscine, and (3*S*)-corynantheidine to their (*3R*)-isomers (left panel), which in turn, are captured by *Mp*SOS to make various spirooxindole pairs (right panel). Error bars demonstrate the standard deviation Sd from 3 biological replicates.

To elucidate the biosynthetic pathways of spirooxindole alkaloids, especially the antiproliferative mitraphylline and its isomers in this species, we integrated the MIA metabolite data with corresponding transcriptomic datasets from all metabolically profiled tissues. As *M. parvifolia* produces the 2 molecules with the same *m/z* ratio and fragmentation patterns of ajmalicine but different retention time (Fig. 5, Supplementary Fig. S14, Supplementary Tables S10 and S11), we speculated that this species has 2 stereoisomers of ajmalicine and therefore would possess enzymes responsible for the formation of both the (3*R*) and (3*S*) isomers of ajmalicine. Furthermore, the abundance and tight correlation between the mitraphylline and isomitraphylline and ajmalicine isomers suggests that they are biosynthetically related, and that there exists a pair of oxidase and reductase enzymes in *M. parvifolia* that converts (3*S*)-ajmalicine and/or its (3*R*)-isomer similar to (*S*) and (*R*)-reticuline in opium poppy (Farrow et al. 2015) (Fig. 6A). Our integrated metabolite and transcriptomic analyses focused on young leaves where spirooxindoles were highly accumulated and the first committed step of MIA pathway *STR* was preferentially expressed (Fig. 5B, Supplementary Figs. S15 and S16).

Using the most highly expressed *strictosidine synthase (MpSTR*) as a bait, several oxidoreductases were identified and hypothesized to be specifically involved in the mitraphylline biosynthetic pathway in *M. parvifolia,* including genes encoding a *FAD-dependent oxidoreductase* (*FAD1*), an *isoflavone reductase* gene (*IR1*), and 5 *CYPs* that were in the CYP71 clan and/or highly correlated with *MpSTR* (*r* > 0.60) (Supplementary Fig. S15). Interestingly, we found that the identified *Isoflavone reductase* belonged to a cluster of *IR*s near the previously identified cluster of *MDR*s on chromosome 12 and all 5 *CYP*s were similarly located in tandem on chromosome 15 (Supplementary Fig. S10). While *FAD1* appeared not to physically cluster with any putative MIA biosynthetic genes, its expression was highly correlated with *STR* and it shared high sequence similarity with a characterized *C. roseus* gene, *O-acetylstemmadenine oxidase* (*CrASO*) indicating potential functional similarity and involvement in MIA biosynthesis. *Cr*ASO catalyzes the oxidation of the vinblastine precursor stemmadenine acetate to precondylocarpine acetate (Caputi et al. 2018; Qu et al. 2019). Thus, we hypothesized that these candidates are involved in mitraphylline biosynthesis in *M. parvifolia*.

To test this hypothesis, we combined all the candidates and transiently co-expressed them in *Nicotiana benthamiana.* Feeding of substrate (3*S*)-ajmalicine (*m/z* 353) to the leaf disks expressing these FADs, IRs, and CYPs candidates led to the formation of mitraphylline and isomitraphylline (*m/z* 369) (Fig. 6A–C, Supplementary Fig. S15), which implies our candidate pool is capable of pushing the pathway from ajmalicine all the way to mitraphylline. Deconvolution of the combination step by step revealed FAD1 and IR1 together can accept (3*S*)-ajmalicine and convert it to a new product with identical *m/z* ratio and MS/MS fragmentation with (3*S*)-ajmalicine substrate (Supplementary Table S10, Supplementary Fig. S14, Supplementary Table S11). In addition, the substrate and enzymatic product eluted at the same retention times with the (3*S*/3*R*)-ajmalicine alkaloids from the *M. parvifolia* extracts (Fig. 6B). This result confirmed the specific involvement of the FAD1 and IR1 enzymes in converting (3*S*)-ajmalicine to its stereoisomer, possibly a (3*R*)-*epi*-ajmalicine in *M. parvifolia in planta* (Fig. 6A). Therefore, the FAD-dependent oxidoreductase and the isoflavone reductase were designated as ajmalicine oxidase (AO) and dehydroajmalicine reductase (DAR). In particular, it is hypothesized that FAD-dependent enzyme *Mp*AO catalyzed the oxidation at C_3_–N bond of the (3*S*)-corynanthe substrate yielded the iminium intermediate. Then, the iminium group was stereo-specifically reduced (hydride addition) at the C_3_ by NADPH-dependent reductase *Mp*DAR to produce the (3*R*)-corynanthe epimers (Fig. 6, A and B) (McDonald et al. 2025).

To test for the substrate specificity of these enzymes, further experiments on *N. benthamiana* with related substrates such as (3*S*)-tetrahydroalstonine, (3*S*)-mayumbine, (3*S*)-yohimbine, (3*S*)-rauwolscine, (3*S*)-corynantheidine were conducted. The combination of *Mp*AO and *Mp*DAR allowed the conversion of these (3*S*)-substrates into their corresponding (3*R*)-epimers (Fig. 6D, Supplementary Figs. S17 to S21). With the MIA substrates containing glucose, such as, strictosidine, strictosamide, and vincosamide, the combination of *Mp*AO and *Mp*DAR could not alter the configuration at C_3_. Thus, the activity of this pair of enzymes was strictly active on the seco-/heteroyohimbane alkaloids (Supplementary Table S12).

With the successful production of (*3R*)-*epi*-ajmalicine, we turned our focus to the cytochrome P450 responsible for the formation of spirooxindole mitraphylline in *M. parvifolia*. Correlation analysis with *MpSTR* also revealed several cytochrome P450 (namely *MpCYP1* to *MpCYP5*) candidates, belonging to the CYP71AU subfamily, co-expressed with *MpSTR, MpAO* and *MpDAR* (Supplementary Fig. S15). These were highly expressed in young leaves and share high sequence similarity with the spirooxindole synthase enzyme, also a CYP71 enzyme (Nguyen et al. 2023). Co-incubation of *Mp*CYP1*, Mp*AO, *Mp*DAR with (3*S*)-ajmalicine ([M + H]^+^  *m/z* 353) as substrate yielded 2 products ([M + H]^+^  *m/z* 369) with an increase of 16 amu, corresponding to an oxidation activity. Multiple reactions monitoring (MRM) scan of the 2 products in UPLC-MS/MS showed the transition of [M + H]^+^ adduct from 369 to 160, characteristic for the oxindole core of the authentic standard mitraphylline and its isomer (Fig. 6C, Supplementary Tables S10 and S11, Supplementary Fig. S14) (Avula et al. 2015; McDonald et al. 2025). The MS/MS spectra of the products were identical to the authentic standards mitraphylline and isomitraphylline (Supplementary Fig. S14). This evidence suggests that *Mp*CYP1 catalyzes the formation of mitraphylline and isomitraphylline (major spirooxindole alkaloids) in *M. parvifolia* from (3*R*)-*epi*-ajmalicine. The spirooxindole formation activity was observed only when *MpAO, MpDAR*, and *MpCYP1* were co-expressed in *N. benthamiana*. The lack of the *Mp*DAR enzyme resulted in no detectable activity of *Mp*CYP1 toward either the dehydroajmalicine intermediate (a proposed product of *Mp*AO) or the (3*S*)-ajmalicine substrate (Fig. 6C). Assay of *Mp*CYP1 with (3*S*)-ajmalicine or other (3*S*) substrates alone did not produce any spirooxindole (Fig. 6C). Thus, (3*R*)-*epi*-ajmalicine, not (3*S*) ajmalicine, is the substrate for *Mp*CYP1. *Mp*CYP1 is a cytochrome P450 enzyme that converts (3*R*)-ajmalicine to their corresponding spirooxindoles mitraphylline and isomitraphylline.

### *Mp*SOS is responsible for the diversity of spirooxindole alkaloids and is distinct for the Cinchonoideae subfamily

Combinatorial assays of *Mp*CYP1, *Mp*AO and *Mp*DAR with related substrates (3*S*)-tetrahydroalstonine and (3*S*)-mayumbine yielded pteropodine alkaloids and mayumbine oxindoles, respectively (Fig. 6, Supplementary Figs. S17 to S18). In addition, assays with *Mp*CYP1 alone converted (3*R*)-hirsutine to the rhynchophylline oxindoles, similar to previously reported spirooxindole synthase in *M. speciosa* (Supplementary Fig. S22) (Nguyen et al. 2023). *Mp*CYP1 was, therefore, designated as *M. parvifolia*  spirooxindole synthase (*Mp*SOS) for its ability to convert several heteroyohimbane and secoyohimbane substrates to their corresponding pentacyclic and tetracyclic spirooxindole scaffolds.

Compared to *Gelsemium*-derived spirooxindole alkaloids featuring a fused oxindole-spirocyclic or bridged/cage-like structure (Chen and Hong 2022), Rubiaceae-derived spirooxindole alkaloids feature a simpler spirocyclic oxindole system. Orthogroup analysis showed that while STR, SGD, MDR are found in various MIA-producing plants, enzymes involved in Rubiaceae-derived spirooxindole are unique to this family. For instance, *SOS* orthologs are only found in *M. speciosa, M. parvifolia, U. rhynchophylla,* and *C. pubescens* (Supplementary Fig. S23). Interestingly, *MpDAR* was only present in *M. parvifolia* (Supplementary Fig. S23), suggesting that the formation of (3*R*)-*epi*-ajmalicine is specific for this species, which produces high amounts of both heteroyohimbanes as well as mitraphylline isomers.

## Discussion

The *Mitragyna* genus is a repository of various pharmaceutically relevant MIAs including spirooxindole alkaloids. Most of research effort on this genus has been focused on *M. speciosa* for its biosynthesis of the psychoactive MIA mitragynine (Shellard 1989; Prozialeck et al. 2012; Kruegel and Grundmann 2018; Han et al. 2020; Kamble et al. 2021; Schotte et al. 2023). Access to the biosynthetic logic of other *Mitragyna* alkaloids is, however, limited due to the lack of omics resources in the species producing divergent MIA scaffolds. Among these understudied species, *M. parvifolia* produces a distinct spirooxindole profile with mitraphylline as a dominant compound, while *M. speciosa* predominantly accumulates mitragynine, speciociliatine, and corynantheidine (Sharma et al. 2019; Leksungnoen et al. 2022; Laforest et al. 2023). In addition to the anticancer potential of mitraphylline, the difference in spirooxindole alkaloid profiles between 2 closely related species presents an excellent opportunity to investigate the genetic basis and biosynthetic diversification of spirooxindoles and MIAs in general. To that end, we constructed a high-quality genome assembly of *M. parvifolia* with high contiguity and completeness as shown by a scaffold N50 of 31.3 Mb and a 98.9% complete BUSCO analysis. Our phylogenomic analyses of the Naucleeae tribe (*M. parvifolia, M. speciosa, U. rhynchophylla*) confirmed a close evolutionary relationship between the *Mitragyna* and *Uncaria* genera, as previously reported based on morphology and barcode sequencing (Razafimandimbison et al. 2004; Löfstrand et al. 2014).

Despite our comprehensive genomic datasets, the longstanding difficulties in resolving the relationships between paleotropical and neotropical lineages of Cinchonoideae (Bremer and Eriksson 2009; Manns and Bremer 2010; Löfstrand et al. 2014; Wikström et al. 2015) remain. In fact, our analysis revealed stronger support for phylogenetic trees in which the Cinchonoideae alliance was not monophyletic (Supplementary Figs. S3 to S4), likely due to the lack of sufficient sampling within and around the Cinchonoideae subfamily. Denser taxon sampling and greater genomic resources will be necessary to resolve this clade.

Our findings suggest that *Cinchona* and *Mitragyna* diverged from their last common ancestor with *Coffea* roughly around the same time based on ortholog *K_s_* distributions, but from each other rapidly thereafter (Fig. 3, Supplementary Table S4). This divergence coincides with the accepted hypothesis that the Cinchonoideae alliance began diversifying during the Oligocene (33.9 to 23 MYA) (Razafimandimbison and Bremer 2001; Wikström et al. 2015).

Our analysis of paralogous gene pairs revealed evidence of a previously unreported contemporaneous polyploidization event shared by *M. parvifolia* and *M. speciosa*, *U. rhynchophylla,* and *C. pubescens*. These duplications appear to have occurred prior to the divergence of the paleotropical Naucleeae tribe and the neotropical Cinchoneae tribe, suggesting they played a critical role in the early evolution of MIA biosynthetic pathways. *Cinchona* produces quinoline MIAs where the quinoline moiety is the pharmacophore common to antimalarial agents (Musiol et al. 2006; Shang et al. 2018; Chen et al. 2021; Yang et al. 2021), while the Naucleeae tribe MIAs belong to the corynanthe-type with the *β*-methoxyacrylate pharmacophore common in fungicides and antiviral agents (Alzeer et al. 2000; Luo et al. 2022). Selective pressures may have resulted in divergent adaptation strategies, ultimately reflected in structurally diverse defense compounds in plants. We show that the *MDR* gene family, responsible for generating the diverse scaffolds of MIAs, underwent periodic diversifying selection as well as expansion throughout its history, supporting this line of thought.

Interestingly, although MDRs with evidence of diversifying selection (Supplementary Fig. S13) and upstream genes involved in MIA biosynthesis were present in *C. eugenioides*, no ortholog of the strictosidine synthase gene was identified in this species, similar to previous findings in *Coffea canephora* (Rai et al. 2021). Thus, loss of the *STR* gene, and therefore the inability to produce strictosidine, the first committed step of MIA biosynthesis, may explain the lack of MIAs in the *Coffea* species and may have precluded this lineage from accessing these scaffolds (Rai et al. 2021).

The evolution of novel scaffolds is a step toward chemical divergence over evolutionary time periods. Gene family analyses highlighted distinct patterns of expansions and contractions that may underline differences in alkaloid profiles between tribes. Notably, very few orthogroups that underwent changes in size were shared in the last common ancestor of the Naucleeae tribe with *C. pubescens*. In addition, major levers involved in plant-pathogen interactions, including response to virus/fungi and sugar/sugar alcohol transport, were enriched in gene families unique to the *Mitragyna* genus (Williamson et al. 2002; Breia et al. 2021).

Taken together, our phylogenomic analyses suggest that a polyploidization event coincided with the radiation of the Cinchonoideae alliance and GO enrichment analysis suggest that unique *Mitragyna* gene families and gene family expansion in *M. parvifolia* are related to adaptation of the tribe to its native range and wet tropical biome. It is widely accepted that WGD events have a major impact on species diversification and ultimately speciation (Muir and Hahn 2015; Zhang et al. 2020b; Zhao et al. 2023). In addition, emerging evidence suggests that WGD and polyploidization events in plants may play a significant role in enabling their adaptation to new biomes (Liddell et al. 2021; Feng et al. 2024). Considering the native ranges of *Mitragyna* species inhabiting swampy tropical forests (Brown et al. 2017), *U. rhynchophylla*, subtropical east Asia (Heitzman et al. 2005), and *C. pubescens* sub-montane to montane rainforests and the complex varied ecosystems of the neotropical realm (Peñarreta et al. 2022), our findings suggest that polyploidization events, and potentially a shared WGD event, in these genera may have facilitated their ability to diversify and colonize such varied environments. Phytochemical diversity via access to divergent MIA scaffolds also plays a significant role in the ecology and evolution of plants.

The Rubiaceae species produce MIAs with highly diverse structures. For instance, corynanthe and oxindole alkaloids are found in *M. speciosa* (kratom) and *Uncaria guanensis* (Cat's claw) (Bindra 1973) (Fig. 1). In *M. parvifolia*, the reduced products of strictosidine aglycone are mostly (3*S*)-ajmalicine and its isomer (3*R*)-*epi*-ajmalicine. Transcriptome and metabolite profiling revealed the significant variations in the developmental regulation of the MIA pathway between *M. parvifolia* and *M. speciosa*. MIAs involved in the formation of mitraphylline from strictosidine were found to accumulate most in young leaves in *M. parvifolia* (Supplementary Table S9), where most mitraphylline biosynthetic genes, namely *MpAO*, *MpDAR*, and *MpSOS* are highly expressed. Recent developments in the model *C. roseus* have also found that gene expression and product accumulation correlate in the same cell types for late-stage alkaloid biosynthesis, namely vindoline and serpentine in leaf idioblasts, although some intracellular transport mechanisms are evident for specific alkaloids (Stander et al. 2020; Li et al. 2023). This type of transport may play an important role in the partitioning of mitraphylline in *M. parvifolia.* The high accumulation of mitraphylline and its isomers, along with their precursors such as strictosidine and ajmalicine, in young leaves, but not in other tissues, suggests that young leaves are the primary site of biosynthesis. The absence of these precursors elsewhere indicates that the end products (mitraphylline and isomitraphylline) are likely transported to other tissues postsynthesis. This is further supported by the strong expression of candidate biosynthetic genes in young leaves compared to other tissues (Fig. 5, Supplementary Figs. S15 to S16). Indeed, the tight correlation of genes encoding enzymes in the same pathway ensures the efficient production of mitraphylline, specific for this species: *Mp*AO, *Mp*DAR, and *Mp*SOS act in concert to enable the biosynthesis of mitraphylline. In particular, the stereochemical inversion of (3*S*)-ajmalicine to its isomer (3*R*)-*epi*-ajmalicine is catalyzed by a pair of oxidase (FAD-dependent, *Mp*AO) and reductase enzymes (*Mp*DAR). This series of reactions seems to be the common strategy to generate new stereoisomers in nature. For example, in the morphine biosynthetic pathway, the conversion of (*S*)-reticuline to (*R*)-reticuline is catalyzed by a fusion enzyme comprising a cytochrome P450 and a reductase module. In this process, (*S)*-reticuline is first oxidized by the P450 domain to form dehydroreticuline, which is then reduced by an aldo-keto reductase domain to yield (*R*)-reticuline (Farrow et al. 2015). *MpAO* and *Mp*DAR shared high amino acid identity with the recently discovered *Ms*CO (corynantheidine oxidase) and *Ms*DCR (dehydrocorynantheidine reductase) enzymes from *M. speciosa*, which converted (3*S*)-corynantheidine to its (3*R*)-epimer (McDonald et al. 2025). *Mp*AO and *Mp*DAR are unique to *M. parvifolia* as they are the only natural pair of enzymes that can convert (*S*) ajmalicine to its (3*R*) isomer. The chiral centers of the polycyclic scaffolds play a key role in the structural diversity of spirooxindoles. The known *Mitragyna* spirooxindole alkaloids are structurally categorized into the secoyohimbane-type (tetracyclic) and the heteroyohimbane-type (pentacyclic). Despite the discovery of numerous tetra- and pentacyclic spirooxindole alkaloids and extensive research on MIA biosynthesis, the enzyme responsible for the oxidative rearrangement of secoyohimbane alkaloids into tetracyclic spirooxindole alkaloids was only identified recently (Nguyen et al. 2023). *Mp*SOS is capable of transforming ajmalicine to its pentacyclic spirooxindole such as mitraphylline and isomitraphylline *in planta,* a biosynthetic step for which no enzyme had previously been functionally characterized. This enzyme also shows high level of versatility as it can accept similar heteroyohimbane molecules, such as (3*S*)-tetrahydroalstonine and (3*S*)-mayumbine with high conversion rate to make various spirooxindole products. With its activities on secoyohimbane substrates such as hirsutine, this *Mp*SOS can produce both pairs of interconvertible stereoisomers tetracyclic and pentacyclic spirooxindoles through intramolecular Mannich reactions (Flores-Bocanegra et al. 2020). Interestingly, these CYPs can only accept (3*R*)-isomers to make interconvertible isomers at C_7_ and C_3_ positions, which raises an interesting question regarding the regulation of their biosynthetic pathway in different species.

*Mp*SOS and its orthologs were only present in the Cinchonoideae subfamily, of which the Naucleeae tribe produces spirooxindole alkaloids. Spirooxindoles have not been reported in *C. pubescens* despite the presence of *SOS* orthologs (Supplementary Fig. S23). There is no evidence of spirooxindole presence in *Ophiorrhiza pumila*, another member of the Rubiaceae family. Such distinct presence strongly suggests that the biosynthetic step catalyzed by SOS enzymes plays a key role in spirooxindole specialization. In addition to having the biochemical capacity, a dynamic interplay of gene evolution, transcriptional regulation, and the repertoire of metabolites ultimately determines the metabolite profile of a given plant.

Altogether, we present a comprehensive look at spirooxindole biosynthesis in *M. parvifolia* and MIA diversification in the Rubiaceae. Our study provides chromosome-level genomic and transcriptomic resources datasets that pave the way for future study of biosynthesis, regulation, and evolution of plant specialized metabolism of the pharmaceutically important *Mitragyna* genus and related plants.

## Materials and methods

### Plant materials

Seeds of *M. parvifolia* used in this study for genomic and transcriptomic analyses were obtained from Horticultural impex, Dehradun, India (https://www.ehorticulture.com/). Plants grown from the seeds were further propagated by cuttings for all the experiments performed in this study. For RNA extraction and metabolite analyses, young leaves (juvenile stage 1), and mature leaves as previously defined, stems, roots, and stipules of *M. parvifolia* were collected and flash frozen in liquid nitrogen, ground and lyophilized (Laforest et al. 2023).

### DNA extraction and sequencing

High-molecular-weight (HMW) genomic DNA was extracted from the fresh young leaves of *M. parvifolia* using a modified SDS extraction protocol. Briefly, nuclei were extracted from young leaf tissue as described previously (Conde et al. 2021), followed by SDS extraction and precipitation of DNA with isopropanol (Loureiro et al. 2007; Mariac 2019; Schalamun et al. 2019). Finally, a salt wash was performed with 5M NaCl to ensure removal of contaminants (Fang et al. 1992). DNA quality and size was confirmed by Qubit 4Flourometer (ThermoFisher Scientific, MA, USA) and TapeStation (Agilent, CA, USA) and sheared to achieve a range of 6 to 20 kb fragments using g-TUBE (Covaris, MA, USA) followed by size selection (>10 kb) using SRE-XS kit (PacBio, USA). Library preparation was performed using the NEBNext companion module for ONT ligation sequencing and ONT ligation sequencing kit V14 (Oxford Nanopore technologies, UK). Long read whole-genome sequencing was performed on the Oxford NanoPore PromethION-24 (Oxford Nanopore Technologies Ltd, Oxford, United-Kingdom) at the University of Florida Interdisciplinary Center for Biotechnology Research (ICBR).

Short read libraries from the same HMW DNA were prepared using NEBNext Ultra II DNA Library Prep Kit for Illumina (NEB kit E7645L). DNA samples were fragmented to 300 to 400 bp using the Covaris E220 Focused-ultrasonicator (Covaris, Cat. No. 500239), as per the manufacturer's instructions, followed by AMPure magnetic bead cleanup (Beckman Coulter, Cat. No. A63881). Sequencing libraries were constructed using the NEBNext UltraTM II DNA Library Prep for Illumina (Cat. No. E7645S) and barcoded using the NEBNext Unique Dual Index Oligos kit (Cat. No. E6440S). Libraries were cleaned, quantified, and sized on the Agilent TapeStation (DNA5000 Screen Tape). Libraries were normalized, pooled, and sequenced on the Illumina NovaSeq X platform to 20 to 30 million reads (2 × 150 nt) per sample.

Chromosome conformation capture and sequencing (Hi-C) was performed using frozen young leaves. Briefly, samples were cross-linked, chromatin DNA was subject to restriction enzyme digestion, biotinylated, and ligated as optimized for plants (Padmarasu et al. 2019). Hi-C libraries were quantified and sequenced on the Illumina (San Diego, CA, USA) Novaseq platform by CD Genomics (Shirley, New York, USA).

### Genome assembly and scaffolding

Genome heterozygosity and ploidy was examined using GenomeScope2 (v2.0) and Smudgeplot (Ranallo-Benavidez et al. 2020) (v0.2.4). *K*-mers of size 21 were generated from Canu (Koren et al. 2017) corrected ONT long-reads using KMC (Kokot et al. 2017) (v 3.2.1). Canu (Koren et al. 2017) (v2.2) was used to assemble the *M. parvifolia* using the following options: -nanopore corMhapFilterThreshold = 0.0000000002 corMhapOptions = “—threshold 0.80 —num-hashes 512 —num-min-matches 3 —ordered-sketch-size 1,000 —ordered-kmer-size 14 —min-olap-length 2,000 —repeat-idf-scale 50 purgeOverlaps = aggressive”. Due to the high resource requirements of Canu, only reads longer than 6,000 bp were used for assembly.

The initial *Canu* assembly was processed through the Purge-Haplotigs pipeline (Roach et al. 2018) (v1.1.3) to produce a haploid assembly which was screened for contamination using the NCBI tool FCS-GX (Astashyn et al. 2024) (v0.5.4). The resulting contig-level assembly was polished with paired and single end Illumina short reads using Pilon (Walker et al. 2014) (v 1.24).

Hi-C paired-end reads were analyzed using the Juicer pipeline with all default settings (Durand et al. 2016a) (v 1.24), to produce inputs for genome scaffolding using the 3D-DNA pipeline (Dudchenko et al. 2017) (v 20170330. Orientation and ordering of contigs were performed with the options “-m haploid -r 1 -i 7000 —editor-repeat-coverage 4 —editor-saturation-centile 10” and all other options as default). The GUI tool Juicebox was used to visualize and manually curate contact maps to improve mis-assemblies (Durand et al. 2016b).

Assembly completeness and contiguity were assessed using BUSCO (Manni et al. 2021a, 2021b) (v 5.2.0) and Quast (Gurevich et al. 2013) (v 5.2.0). Syntenic relationships between chromosomes were determined using MCScanX (Wang et al. 2024) (v20130328) and visualized using Circos (Krzywinski et al. 2009) (v 0.69-9).

### RNA-sequencing and transcriptome assembly

High-quality total RNA was isolated from *M. parvifolia* leaf developmental stages (Supplementary Fig. S24), roots, stems, and stipules in 3 biological replicates using CTAB RNA extraction protocol (Brose et al. 2021). RNA integrity and quantity were assessed using the RNA 6,000 Nano kit (Bioanalyzer 2100) and Qubit 4 Fluorometer (Life Technologies). Library construction was performed at UF ICBR Gene Expression Core using NEBNext Poly(A) mRNA magnetic isolation module (New England Biolabs, catalog #E7490) and the NEBNext Ultra II Directional RNA library prep kit (New England Biolabs, catalog #E7760) with 400 ng RNA per sample for library preparation, fragmentation, adaptor ligation, and library amplification. Equimolar polled libraries were sequenced on an Illumina NovaSeq X Plus (2 × 150 nt) at the University of Florida ICBR NextGen Sequencing facility. Read quality was evaluated using FastQC (Andrews 2010) (v 0.11.9) and MultiQC (Ewels et al. 2016) (v1.7). Adaptors and low-quality reads were trimmed and filtered using Trimmomatic (Bolger et al. 2014) (v0.36). The *M. parvifolia* transcriptome was assembled *de novo* using Trinity's (vr20140413) genome guided approach (Grabherr et al. 2011), with a max intron length of 20000 based to better partition paralogous genes and capture sequence variations in RNA-seq libraries (Brose et al. 2021). Paired RNA-seq end reads passing QC were aligned to the final assembly with HISAT2 (Kim et al. 2019) (v2.2.1), bam files merged and sorted, and the final file was provided to Trinity for *de novo* transcriptome assembly (Fu et al. 2012) and transcriptome completeness was assessed using BUSCO.

### Genome annotation and quality evaluation

*De novo* TE annotation was carried out using the EDTA (Ou et al. 2019) (v2.1.0) pipeline with the –sensitive option set to 1. The resulting TE library was used to generate a soft masked genome using bedtools (Quinlan and Hall 2010) (v2.30.0). The scaffolded genome assembly was annotated using the Maker pipeline (Cantarel et al. 2008), with curated TE libraries generated above provided for masking of repeat elements and the *de novo* assembled transcriptome as evidence for training the Exonerate (Slater and Birney 2005), SNAP (Korf 2004), and Augustus (Stanke et al. 2008) gene predictors. The completeness of the annotation was assessed using BUSCO (Manni et al. 2021a). Functional annotation was carried out using eggnog-mapper (Cantalapiedra et al. 2021) and Pannzer (Törönen and Holm 2022). A species-specific GO database was generated using the AnnotationForge (Carlson and Pagès 2017) R package and GO enrichment analysis was carried out with Clusterprofiler (Wu et al. 2021).

### Differential gene expression analysis and GO enrichment analysis

High-quality (QC > 30) and paired reads were used to align to the *M. parvifolia* genome using HISAT2 (Kim et al. 2019) (v 2.2.1). Transcript abundance was estimated using htseq-count (Anders et al. 2015) (v 2.0.0). Replicate homogeneity was checked using a multidimensional scaling analysis with the R package edgeR (v3.36.0). Transcripts across samples were normalized using TMM normalization to control variations in library size. For each species, samples were compared pairwise for differential expression using edgeR (Robinson et al. 2010). Genes with a log_2_-fold change of ±1 and FDR adjusted (<0.05) *P*-value < 0.05 were marked as differentially expressed genes (DEGs).

### Phylogenomic, gene family contraction/expansion, and WGD analysis

Orthogroups for all species compared were identified using the Orthofinder (Emms and Kelly 2019) (v2.5.5), with amino acid sequences from proteomes of species provided in Supplementary Table S3 using default settings. Astral-Pro3 (Zhang et al. 2020a) was used to infer a maximum likelihood phylogenetic tree placing *M. parvifolia* in the published phylogeny (Razafimandimbison and Bremer 2001; Wikström et al. 2015; Kumar et al. 2022) using gene families shared between all species studied and the option -c. Divergence time was estimated based on TimeTree5 (Kumar et al. 2022) database (125 MYA for Node 0) for CAFÉ5 (De Bie et al. 2006) (v5.0.0) to estimate gene family expansion and contraction. Gene families that have undergone expansion or contraction were further analyzed for GO annotation and enrichment using ClusterProfiler (Wu et al. 2021). Synonymous substitution rates per gene (Ks) were evaluated for orthologs within Rubiaceae to estimate divergence time between species using KsRates (Sensalari et al. 2022) (v 1.1.1). Paralogous gene predictions, *K_s_* value calculations between paralogs for each species to evaluate WDGs, and classification of duplications were done using MCScanX (Wang et al. 2024). Macrosyntenic relationships between Rubiaceae genomes were determined and visualized using the JCVI (Tang et al. 2024). MCScan tool. BUSTED (Murrell et al. 2015) and ABSREL (Smith et al. 2015) analyses were carried out on orthogroups using hyphy (Pond et al. 2005) to assess selective pressure on MIA pathway genes across species over time. Alignment and Newick files for supplemental cladograms can be found in Supplementary Data Set 1, and Supplementary Objects S1 and S2.

### Chemical reagents

Major kratom alkaloids, mitragynine, speciociliatine, speciogynine, mitraciliatine, corynantheidine, paynantheine, isopaynantheine, isocorynantheidine, corynoxine, speciofoline, and, mitraphylline (purity ≥98%) were isolated in-house from kratom alkaloid rich extract and characterized using carbon nuclear magnetic resonance (^13^C NMR), proton nuclear magnetic resonance (^1^H NMR), and high-performance liquid chromatography-photometric diode array-quadrupole time of flight (HPLC−PDA-QToF) mass spectrometry (Sharma et al. 2019; Laforest et al. 2023). Ajmalicine and corynoxine-B were procured from Cayman Chemicals (Michigan, USA). Hirsutine was acquired from Sigma Aldrich Inc. (Missouri, USA). Mayumbine, tetrahydroalstonine and isomitraphylline were purchased from Arctom (California, USA). Strictosidine standard was kindly donated by Dr. Yi Tang (University of California, LA, USA) (Misa et al. 2022). 7-Hydroxymitragynine and 9-hydroxycornantheidine (purity ≥98%) were semi-synthesized from mitragynine in-house and characterized with other alkaloids (Ponglux et al. 1994; Chiang et al. 2024).

### Alkaloid extractions, identification, and quantitation by LC-MS/MS

Alkaloid extractions were performed as previously optimized using 50 mg of frozen ground tissue (Laforest et al. 2023). The identification and quantification of targeted alkaloids were carried out utilizing a Waters Acquity Class I ultraperformance liquid chromatography (UPLC) system paired with a XevoTQ-S Micro triple quadrupole mass spectrometer (Milford, MA, USA) using an Acquity BEH C18 column at a flow rate of 0.35 mL/min as previously described (Sharma et al. 2019; Laforest et al. 2023). The detection was performed in MRM method with electron spray ionization operating in positive ionization mode monitoring the transitions (Supplementary Tables S13 and S14).

Statistical analysis was performed using a mixed model in SAS JMP to compare means alkaloid content across different tissue types (Supplementary Data Set 2). Least Squared Means estimates with Student's *t*-test for initial comparison was used for multiple comparisons on SAS JMP. Alkaloid quantities below the lower level of quantification were imputed as a random value between 0 and lower level of quantification for statistical analysis.

### Candidate genes selection

Based on the highest accumulation of strictosidine in the young leaves, the genes annotated as *STR* were selected, and the candidate with the highest expression level in young leaves was chosen for further analyses. The correlation analysis (Pearson correlation) and hierarchical clustering using *M. parvifolia* transcriptome and *MpSTR* as key gene were performed by R (R studio 2024.04.2). Log2-transformed CPM values for each transcript in multiple tissues were scaled by using min-max normalization from 0 to 1 for clustering visualization. The oxidoreductase candidates clustering with *MpSTR* were selected for activity screening.

### Candidate genes cloning and transformation of GV3101 *Agrobacterium tumefaciens* cells

Coding regions of the *M. parvifolia* candidate genes were obtained from the *de novo* transcriptome. Synthetic gene candidates (Twist Bioscience, USA) (Supplementary Table S15) were inserted into the *Age*I and *Xma*I restriction sites of the pEAQ-HT plasmid (Sainsbury et al. 2009) by using 5× In-Fusion cloning system (Takara Bio USA Inc.). The crude reaction was transformed into Stellar *E. coli* cells (Takara Bio USA Inc.) and spread on the LB agar plate with 50 *µ*g/mL kanamycin antibiotic. Plasmids of positive transformants (approx. 200 ng/µL) were mixed with GV3101 *Agrobacterium tumefaciens* cells and electroporated (VWR) at 2.5 kV. Postelectroporation, cells were mixed with 150 *µ*L of SOC media and incubated at 28 °C with agitation for 2 h before plating on the LB agar plates with kanamycin (50 *µ*g/mL), gentamycin (30 *µ*g/mL), and rifampicin (25 *µ*g/mL).

### Transient expression in *Nicotiana benthamiana* and UPLC-MS/MS analyses

The single colony of transformed *A. tumefaciens* was selected and inoculated in 10 mL LB + kanamycin (50 *µ*g/mL), gentamycin (30 *µ*g/mL), and rifampicin (25 *µ*g/mL) for 48 h. The cells were collected by centrifugation and resuspended in 1 mL infiltration buffer pH 5.6 (10 mm 2-(N-morpholino) ethanesulfonic acid (MES), 10 mm MgCl_2_, 200 *µ*M 3′,5′-dimethoxy-4′-hydroxyacetophenone (acetosyringone). The cells were incubated under light-free condition for 2 to 3 h. The OD_600_ of each mixture was measured, and the strains were pooled, with each strain having a final OD_600_ of 0.4. Approximately 1 mL of mixed cells were infiltrated into the abaxial side of one *N. benthamiana* leaf (3-wk-old plants grown under 16-h light and 8-h dark conditions). After 3 d, the 5-mm leaf disks were collected from the infiltrated leaves and incubated with 200 *µ*L of 50 *µ*M substrates for 24 h. After the incubation, the leaf disks were ground in liquid nitrogen and extracted with 85% methanol + 0.1% formic acid solution. The extracted solution was filtered through the 0.2 *µ*m syringe filter and subjected to the UPLC coupled with a Xevo TQ-S Cronos Triple Quadrupole Mass Spectrometer for product detection. The UPLC-MS/MS analyses were carried out on an XBridge BEH XP (10 × 2.1 mm, 1.7 mm) column at a 0.6 mL.min^−1^ flow rate. The column was pre-equilibrated in 90% solvent A (water + 0.1% formic acid), and 10% solvent B (acetonitrile + 0.01% formic acid). The eluting program was: 0 to 8 min, 10% to 50% B; 8.0 to 8.5 min, 50% to 100% B; 8.5 to 9.5 min, 100% B; and 9.5 to 11 min, 100% to 10% B to equilibrate the column. The Selected Ion Recording (SIR) and MRM modes were preselected to specifically detect the MIAs and spirooxindole alkaloids (Supplementary Table S10). Dwell time of 25 ms was applied to each MRM transitions. The daughter scans for MS/MS spectra acquisition were conducted in positive ESI mode, mass range m/z 50-387, scan time 0.1 min, cone value 76 V, collision energy (CE) 15 V.

### Accession numbers

Sequence data from this article can be found in the GenBank/EMBL data libraries under accession numbers provided in Supplementary Data Set 3.

## Supplementary Material

koaf207_Supplementary_Data

## Data Availability

Data supporting the findings of this work are available within the paper and its Supplementary Information Files. The datasets and plant materials generated and analyzed during the current study are available from the corresponding authors upon reasonable request. DNA ONT long-reads and Illumina short reads (SAMN46901897), Illumina RNA short reads (SAMN46926934-SAMN46926948), and the genome assembly have been deposited in the NCBI Sequence Read Archive (SRA) database under BioProject PRJNA1225746 (Supplementary Data Set 4). The Gene Ontology (GO) database is available upon reasonable request to corresponding authors.

## Supplementary data

The following materials are available in the online version of this article.

**Supplementary Figure S1.** Genomic characteristics of *Mitragyna parvifolia* genome.

**Supplementary Figure S2.** Distribution of scaffold size after scaffolding *M. parvifolia* genome assembly with Hi-C.

**Supplementary Figure S3.** Single copy ortholog IqTree species cladogram.

**Supplementary Figure S4.** OrthoFinder inferred speies cladogram.

**Supplementary Figure S5.** Peak identification in *K_s_* values calculated for paralogous gene pairs for each species.

**Supplementary Figure S6.** Types of duplications between paralogous gene pairs identified in *M. parvifolia*

**Supplementary Figure S7.** Gene depth histogram per species comparison showing a 1:2 ratio between *Coffea eugenioides* and members of Cinchonoideae.

**Supplementary Figure S8.** Upset plots indicating the largest 15 orthogroups unique to Rubiaceae species in this study.

**Supplementary Figure S9.** GO enrichment analysis of expanded gene families.

**Supplementary Figure S10.** Ideogram showing distribution of putative MIA genes across the *M. parvifolia* genome.

**Supplementary Figure S11.** Macrosyntenic comparison of Rubiaceae family species included in this study with chromosome-level genome assemblies.

**Supplementary Figure S12.** Cladogram depicting significant gene expansion and contraction in the medium-chain reductase gene family.

**Supplementary Figure S13.** Cladogram of MDR gene family, and ASBERL results indicating diversifying selection on branches of Gentianales specific clade.

**Supplementary Figure S14.** Daughter scans and MS/MS profile of biochemical assays with (3*S*)-ajmalicine substrate.

**Supplementary Figure S15.** Hierarchical clustering of MIA biosynthetic genes in *M. parvifolia.*

**Supplementary Figure S16.** Expression patterns of MIA biosynthetic pathway in *M. parvifolia*.

**Supplementary Figure S17.** Biochemical characterization of *Mp*AO, *Mp*DAR and *Mp*SOS in *N. benthamiana* with (3*S*)-tetrahydroalstonine substrate.

**Supplementary Figure S18.** Biochemical characterization of *Mp*AO, *Mp*DAR and *Mp*SOS in *N. benthamiana* with (3*S*)-mayumbine substrate.

**Supplementary Figure S19.** Biochemical characterization of *Mp*AO, *Mp*DAR and *Mp*SOS in *N. benthamiana* with (3*S*)-yohimbine substrate.

**Supplementary Figure S20.** Biochemical characterization of *Mp*AO, *Mp*DAR and *Mp*SOS in *N. benthamiana* with (3*S*)-rauwolscine substrate.

**Supplementary Figure S21.** Biochemical characterization of *Mp*AO, *Mp*DAR and *Mp*SOS in *N. benthamiana* with (3*S*)-corynantheidine substrate.

**Supplementary Figure S22.** Biochemical activity *MpSOS* in *N. benthamiana* with substrate (3*R*)-hirsutine.

**Supplementary Figure S23.** Distribution of MIA biosynthetic enzyme gene families in different species.

**Supplementary Figure S24.** Examples of *Mitragyna parvifolia* young (left) and mature (right) leaves.

**Supplementary Table S1.** Resources used in *M. parvifolia* genome sequencing and assembly statistics.

**Supplementary Table S2.**  *M. parvifolia* Repeat annotation classes and content.

**Supplementary Table S3.** List of genomic resources of plant species used in comparative phylogenomic analyses in this study.

**Supplementary Table S4.** Adjusted *K_s_* distributions between *M. parvifolia* and Rubiaceae orthologs.

**Supplementary Table S5.** Top 25 biological processes and molecular functions enriched in unique *Mitragyna parvifolia* Orthogroups.

**Supplementary Table S6.** Top 25 biological processes and molecular functions enriched in expanded *Mitragyna parvifolia* Orthogroups.

**Supplementary Table S7.** Top 25 biological processes and molecular functions enriched in Orthogroups unique to *Mitragyna* species.

**Supplementary Table S8.** Top 25 biological processes and molecular functions enriched in Orthogroups expanded in the Naucleeae tribe.

**Supplementary Table S9.** MIAs detected in *M. parvifolia* tissues.

**Supplementary Table S10.** MRM transitions, cone values, collision energy of alkaloids analyzed in this study for transient expression in *Nicotiana benthamiana*.

**Supplementary Table S11.** Key fragmentation data from MS/MS spectra of enzymatic products in this study.

**Supplementary Table S12.** Substrate specificity of the combination of *Mp*AO and *Mp*DAR enzymes.

**Supplementary Table S13.** Mass spectrometer compound parameters for alkaloids and internal standard (IS) used for quantitation.

**Supplementary Table S14.** Mass spectrometer electrospray ionization (ESI) source parameters for identification and quantitation of alkaloids.

**Supplementary Table S15.** Sequences of tested candidates in this study.

**Supplementary Data Set 1.** Newick and MSA corresponding to cladograms presented in this work.

**Supplementary Data Set 2.** Effects test results for *M. parvifolia* alkaloid quantitation.

**Supplementary Data Set 3.** Gene accessions referred to in this work.

**Supplementary Data Set 4.** Accessions of sequencing reads generated in this work.

**Supplementary Object S1.** Single copy ortholog MSA.

**Supplementary Object S2.** Orthofinder species tree MSA.

## Dive Curated Terms

The following phenotypic, genotypic, and functional terms are of significance to the work described in this paper:

CYP71 Gramene: AT3G44600
CYP71 Araport: AT3G44600

alkaloid CHEBI: CHEBI:22315

dna Gramene: EO:0007532
dna Araport: EO:0007532

TMM Gramene: Method in EdgeR to normalize read counts (trimmed mean of M values)
TMM Araport: Method in EdgeR to normalize read counts (trimmed mean of M values)

ONT Gramene: oxford nanopore technologies
ONT Araport: oxford nanopore technologies

ajmalicine CHEBI: CHEBI:2524

NCBI Gramene: https://www.ncbi.nlm.nih.gov
NCBI Araport: https://www.ncbi.nlm.nih.gov
