# Supplementary material for: A chromosome-level Mitragyna parvifolia genome unveils spirooxindole alkaloid diversification and mitraphylline biosynthesis
Source: Plant Cell. 2025 Aug 18;37(9):koaf207. doi: 10.1093/plcell/koaf207 (PMC12419693; doi:10.1093/plcell/koaf207)
Supplement: koaf207_Supplementary_Data [file koaf207_supplementary_data.zip › Supplementary Figures_Revised.pdf]

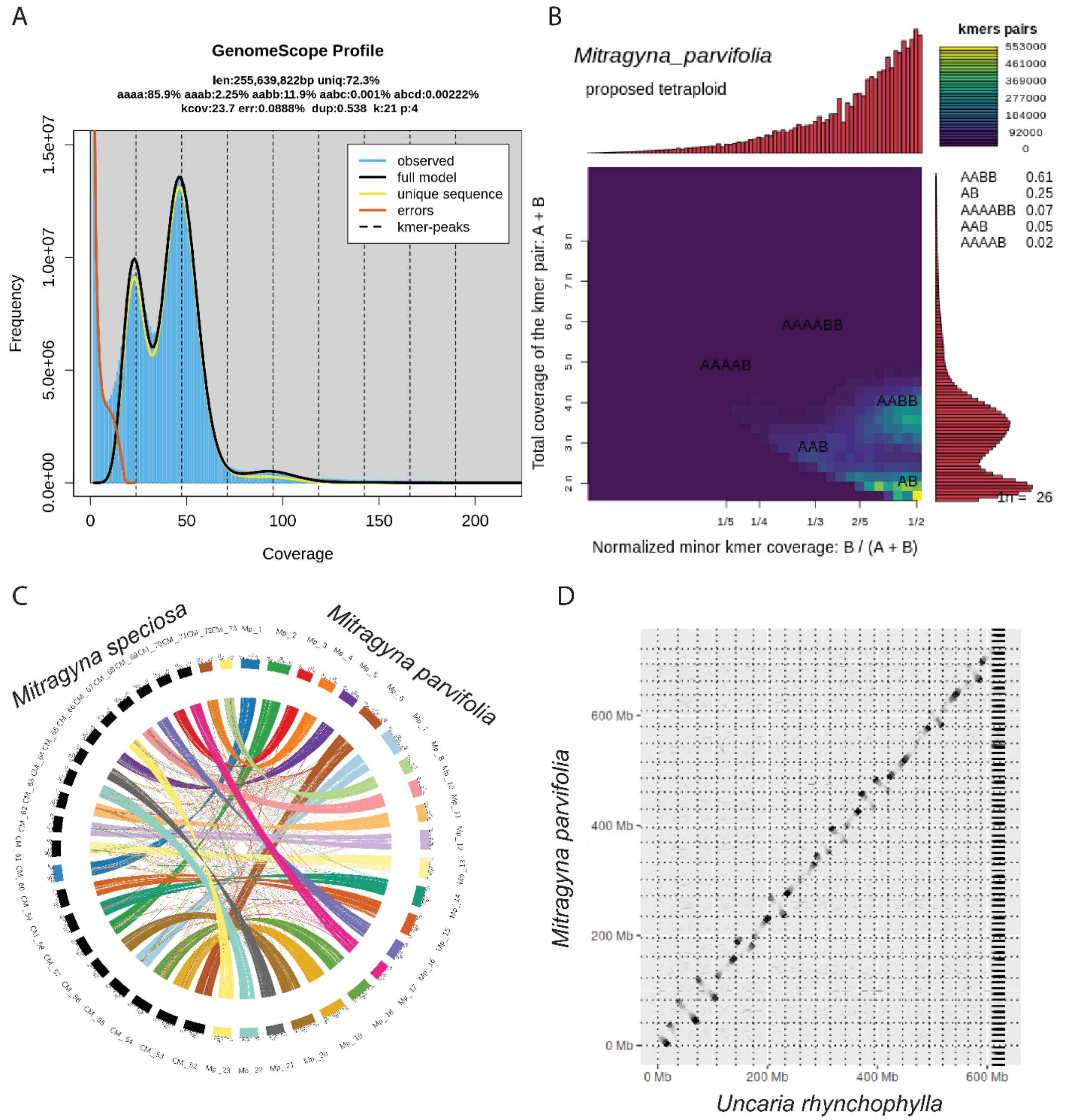

Supplementary Figure S1: Genomic characteristics of *Mitragyna parvifolia* genome A) Genomescope *K*-mer spectra analysis of *M. parvifolia* Canu-corrected ONT long reads, showing estimated genome size (1x), heterozygosity, coverage, and B) SmudgePlot estimated genome structure. C) Collinearity with chromosome scale *M. speciosa* genome assembly. D) Dotplot depicting collinearity between *Uncaria rhynchophylla* and *M. parvifolia.* Related to Figure 2.

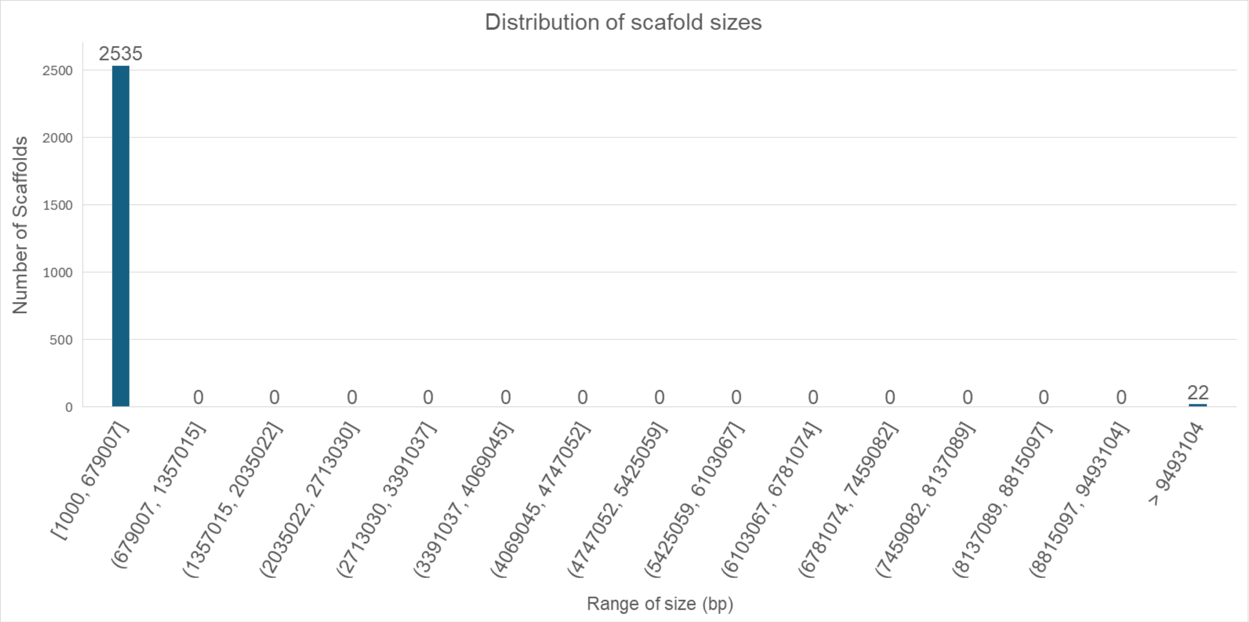

Supplementary Figure S2: Distribution of scaffold size after scaffolding with Hi-C showing 22 chromosome length scaffolds. Related to Figure 2. 1.6 percent of BUSCO genes were found in unanchored scaffolds (2535), whereas the 22 largest scaffolds contained 98.9% of complete BUSCO genes.

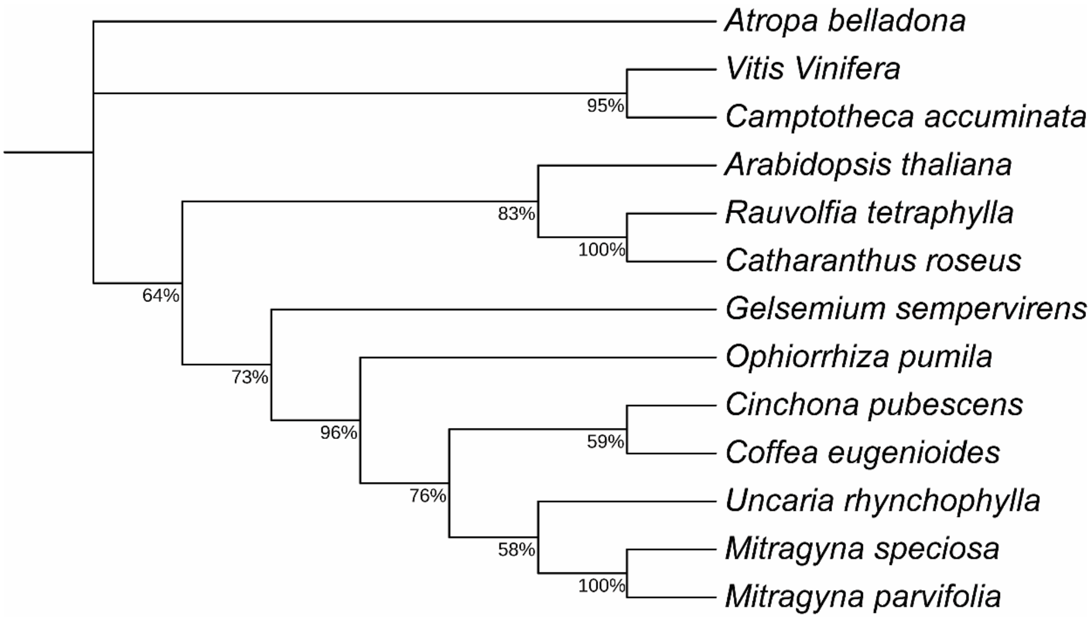

Supplementary Figure S3: Single copy ortholog IqTree cladogram with bootstrap values. Related to main Fig. 3.

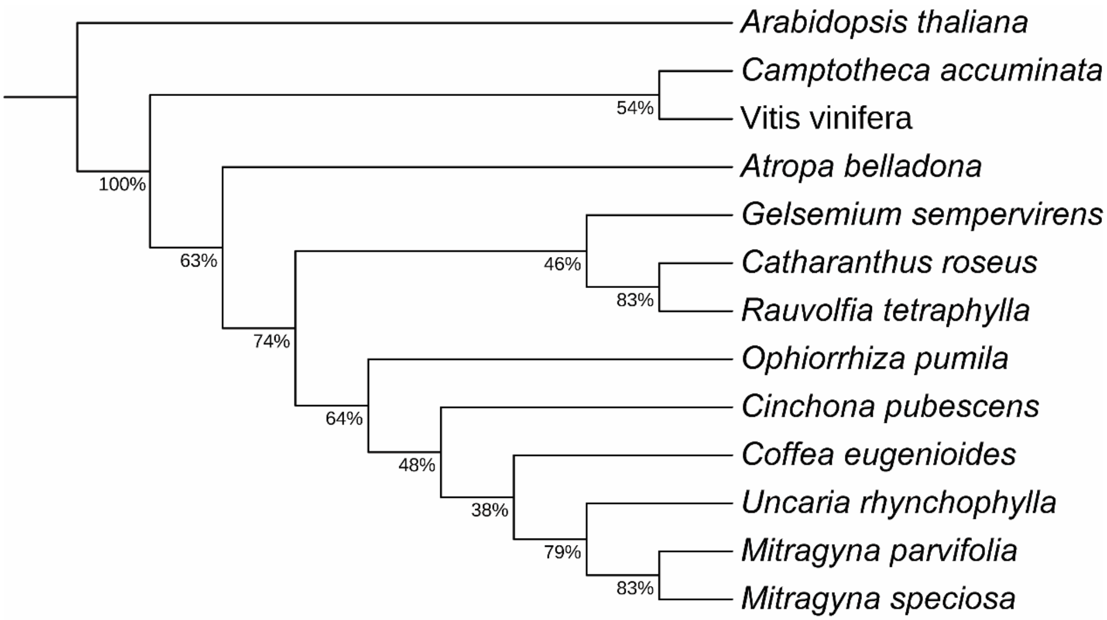

Supplementary Figure S4: OrthoFinder inferred cladogram showing incorrect topology (Cinchonoideae is paraphyletic), bootstrap values are shown for branches. Related to main Fig. 3.

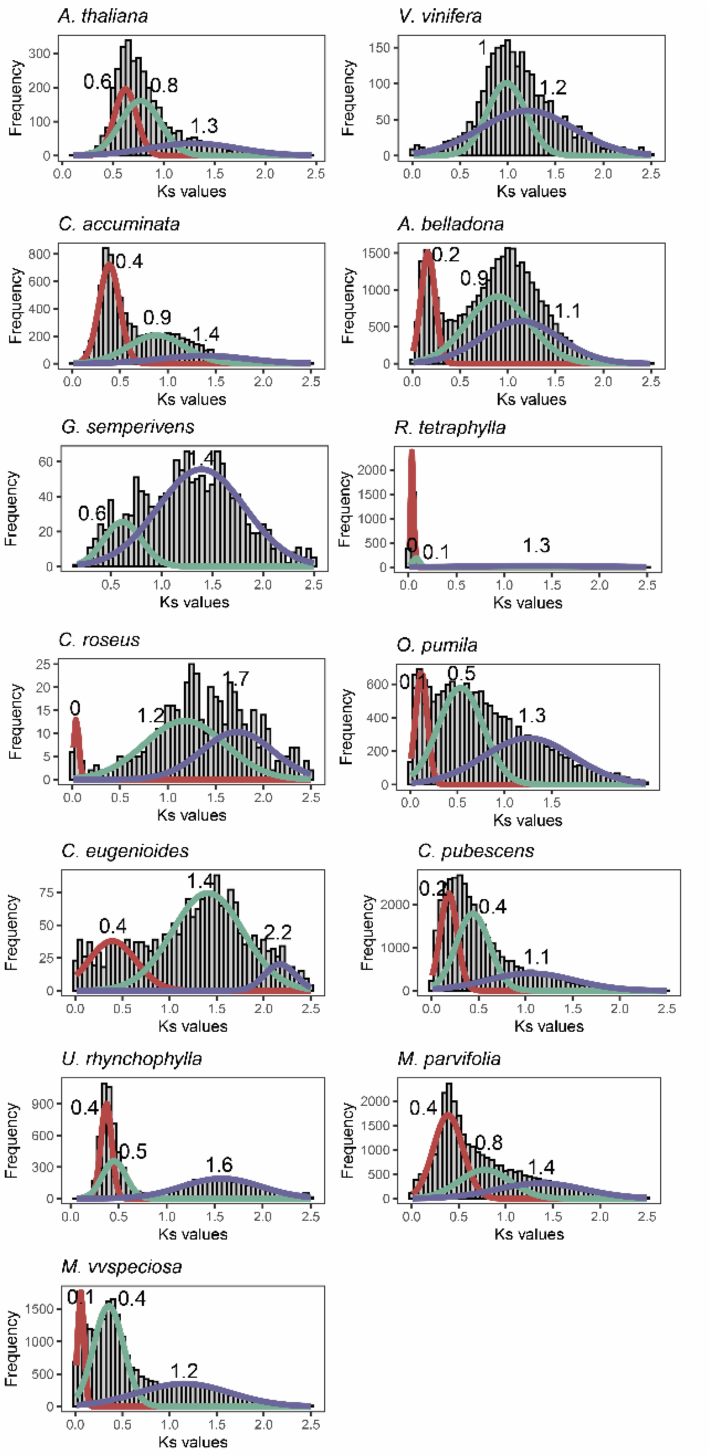

Supplementary Figure S5: Peak identification in Ks values <2.5 calculated for paralogous gene pairs for each species. Each line (red, teal, purple) represents a peak identified by the find_ks_peaks function of the DoubleTrouble package in R. Related to main Fig. 3.

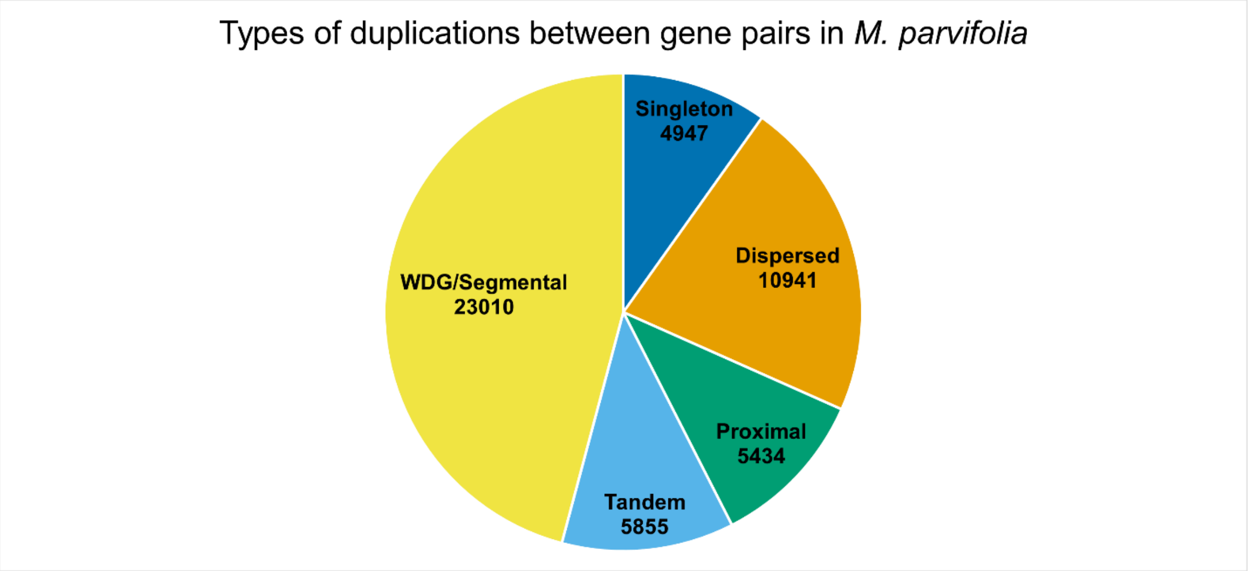

Supplementary Figure S6: Types of duplications between paralogous gene pairs identified in *M. parvifolia*. Related to main Fig. 3.

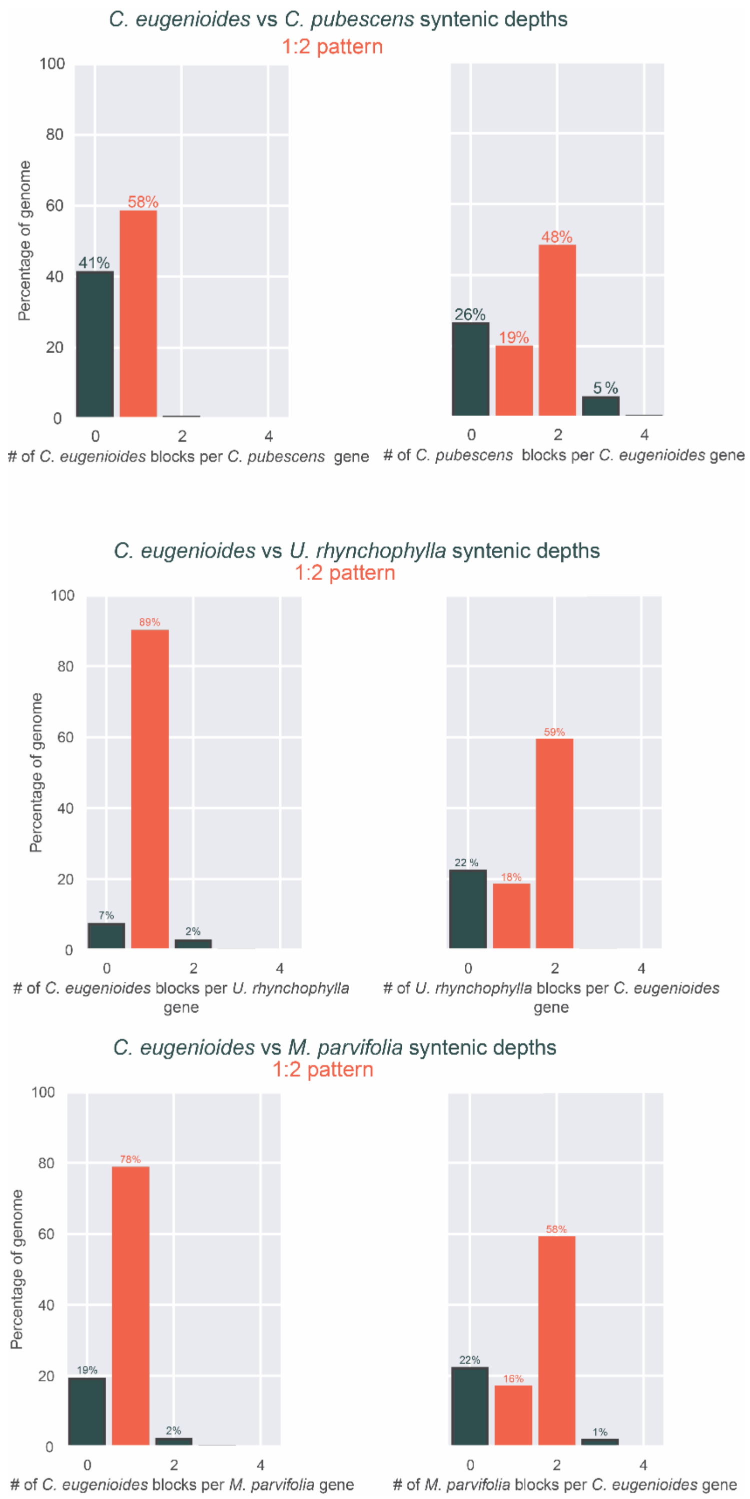

Supplementary Figure S7: Gene depth histogram per species comparison showing a 1:2 ratio between *Coffea eugenioides* and all members of Cinchonoideae. The percentage of genes participating in the calculated syntenic depth (1:2 pattern) are highlighted in orange, from 1 to the peak. Related to main Fig. 3.

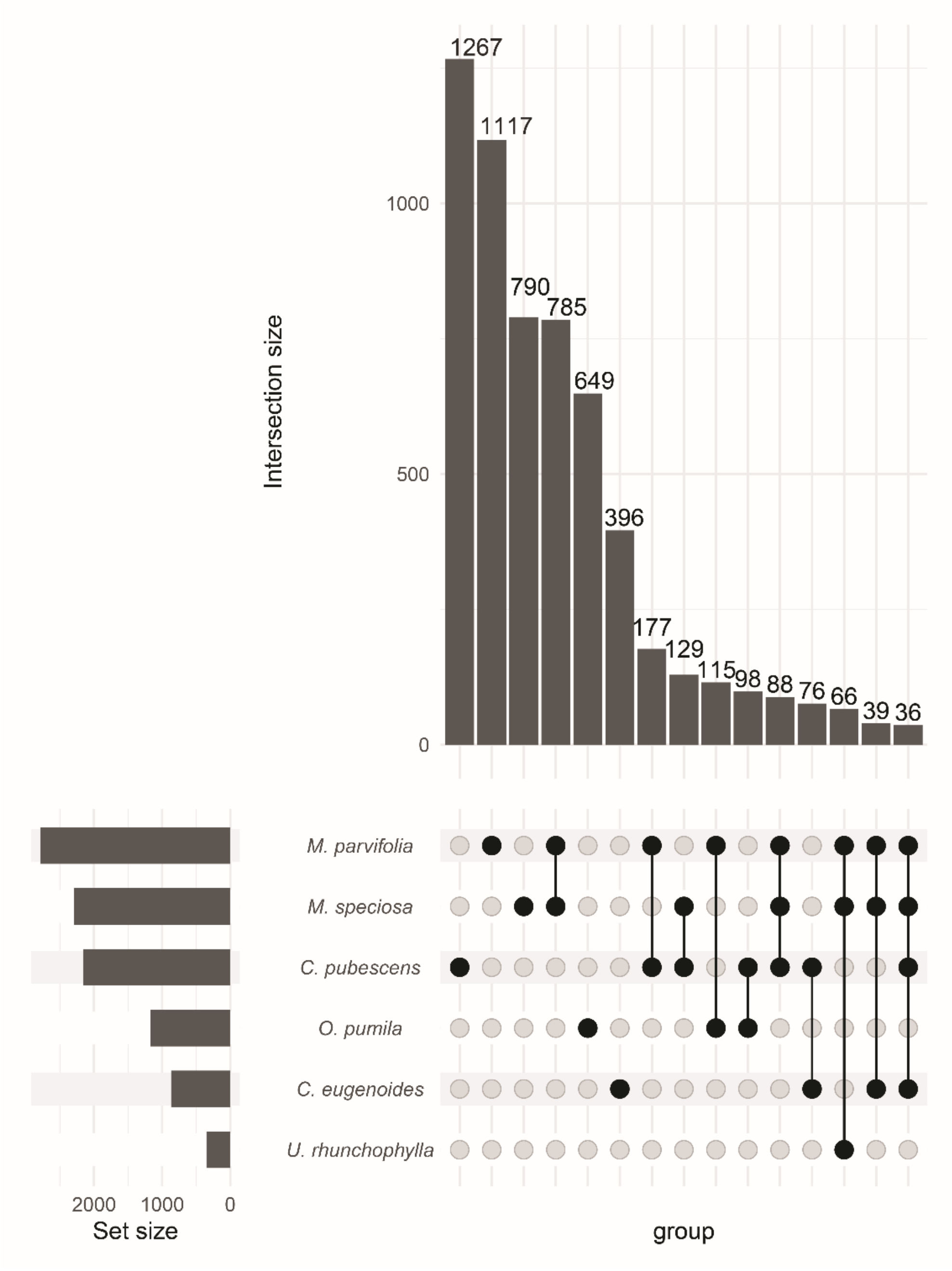

Supplementary Figure S8: Upset plots indicating the largest 15 orthogroups unique to the Rubiaceae family. *M. parvifolia* and *M. speciosa* have the second and third largest number of unique gene families after *C. pubescens*. Related to main Fig. 3.

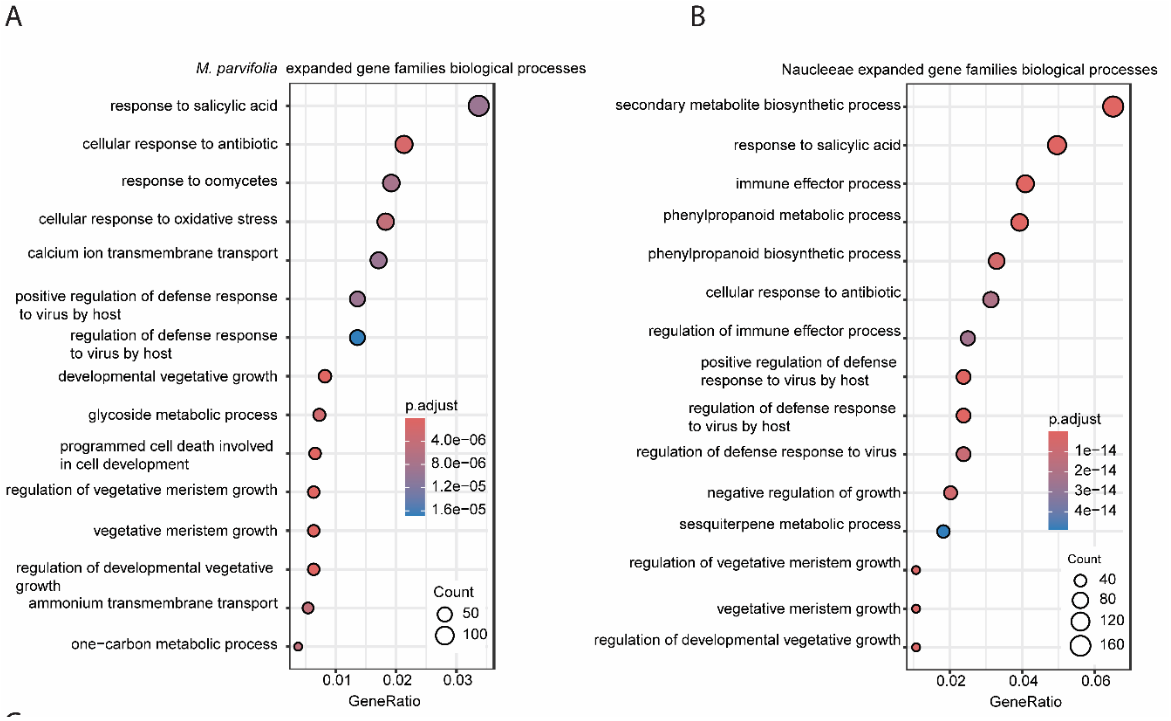

Supplementary Figure S9: GO enrichment analysis of expanded gene families. A) enriched biological processes in *M. parvifolia* expanded orthogroups, and B) Orthogroups expanded in the Naucleeae tribe. Related to main Fig. 3.

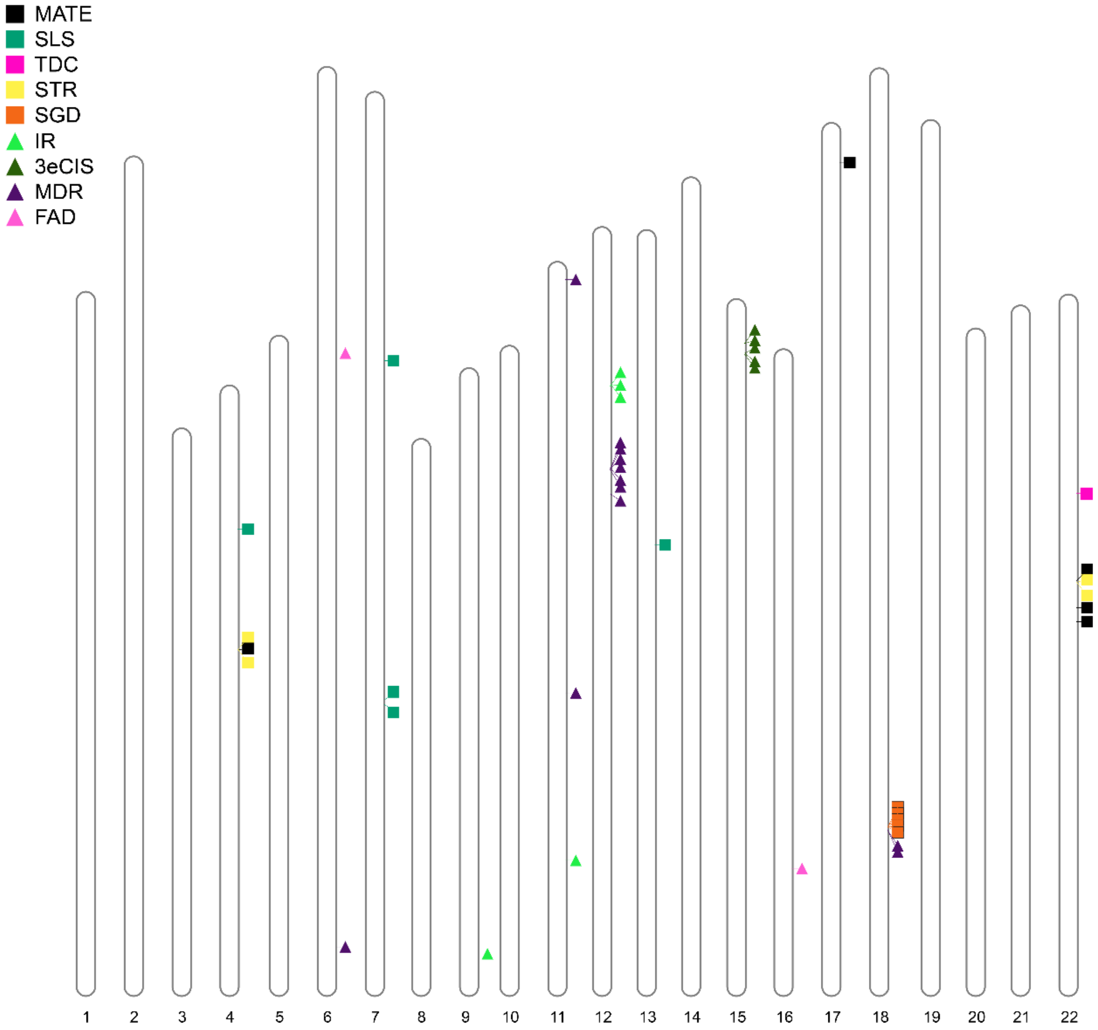

Supplementary Figure S10: Ideogram showing distribution of putative MIA genes across the M. *parvifolia* genome. MATE: multidrug and toxic compound extrusion, SLS: secologanin synthase, TDC: tryptophan decarboxylase, STR: strictosidine synthase, SGD: strictosidine β-D-glucosidase, IR: Isoflavone reductase, 3eCIS: 3-epi-corynoxeine/isocorynoxeine synthase, MDR: medium chain reductase, FAD: FAD-dependent oxidase.Related to main Fig.4.

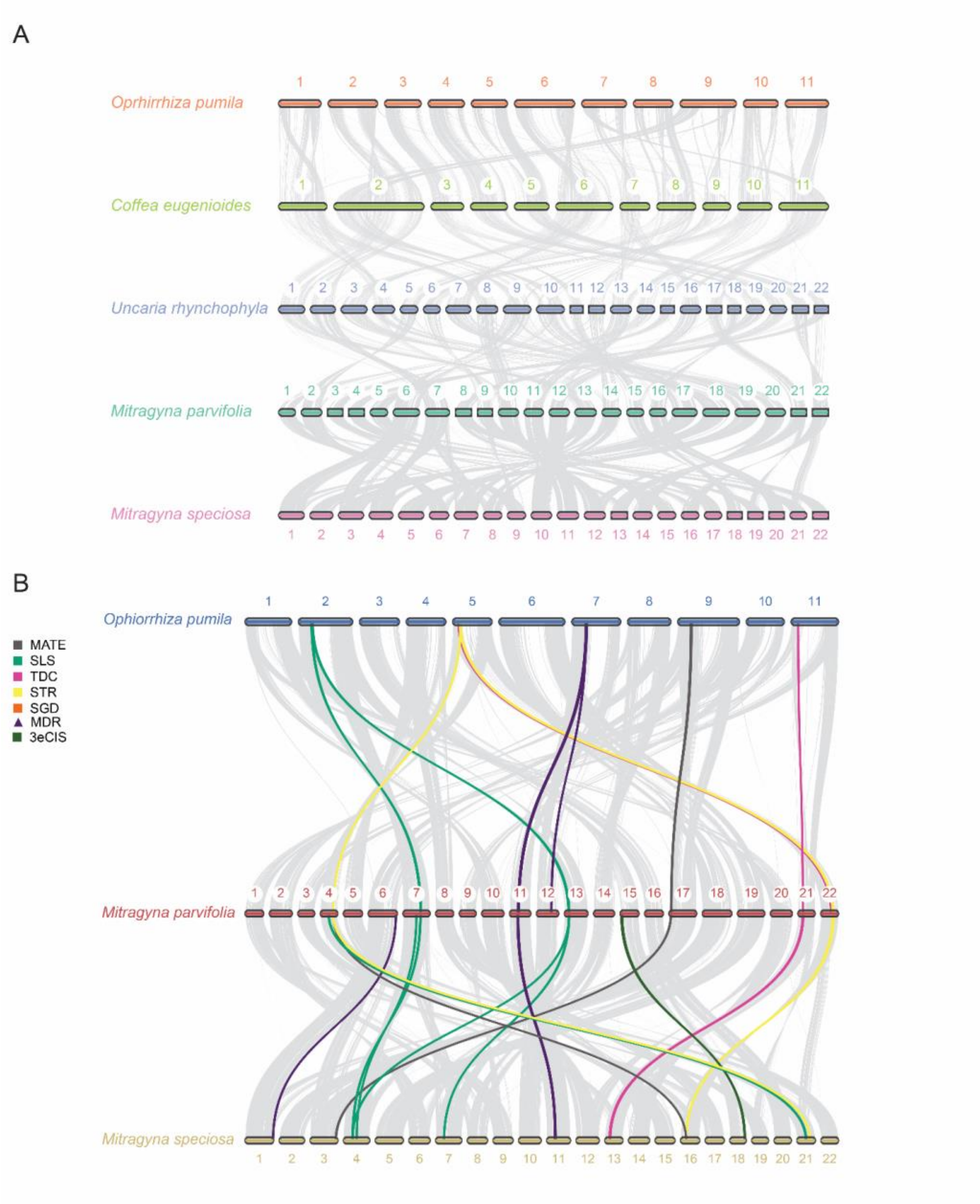

Supplementary Figure S11: Macrosyntenic comparison of Rubiaceae family species included in this study with chromosome level genome assemblies A) Synteny between all species. B) Pairwise macrosyntenic analysis of *Mitragyna parvifolia, Ophirrhiza pumila*, and *Mitragyna speciosa* with putative MIA biosynthetic genes highlighted. MATE: multidrug and toxic compound extrusion, SLS: secologanin synthase, TDC: tryptophan decarboxylase, STR: strictosidine synthase, SGD: strictosidine β-D-glucosidase, MDR: medium chain reductase, 3eCIS: 3-epi-corynoxeine/isocorynoxeine synthase.

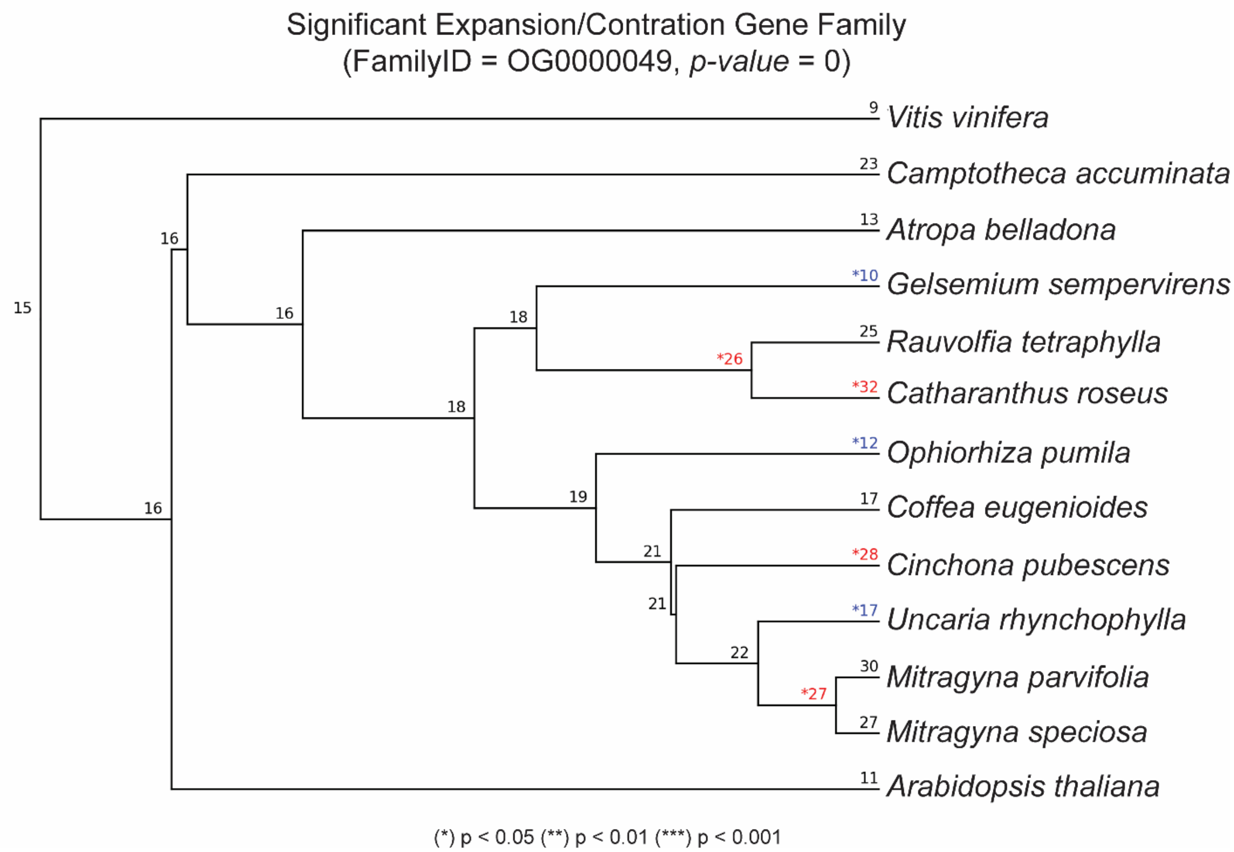

Supplementary Figure S12: Cladogram depicting significant gene expansion and contraction in the medium chain reductase gene family. Significant changes are marked by *. Numbers represent the total number of genes. CAFE computes p-values between an ancestral state and a branch based on Viterbi and branch-cutting methods as described in De Bie et al., *Bioinformatics*, Volume 22, Issue 10, 2006, Pages 1269–1271. Related to main Fig. 3 and 4.

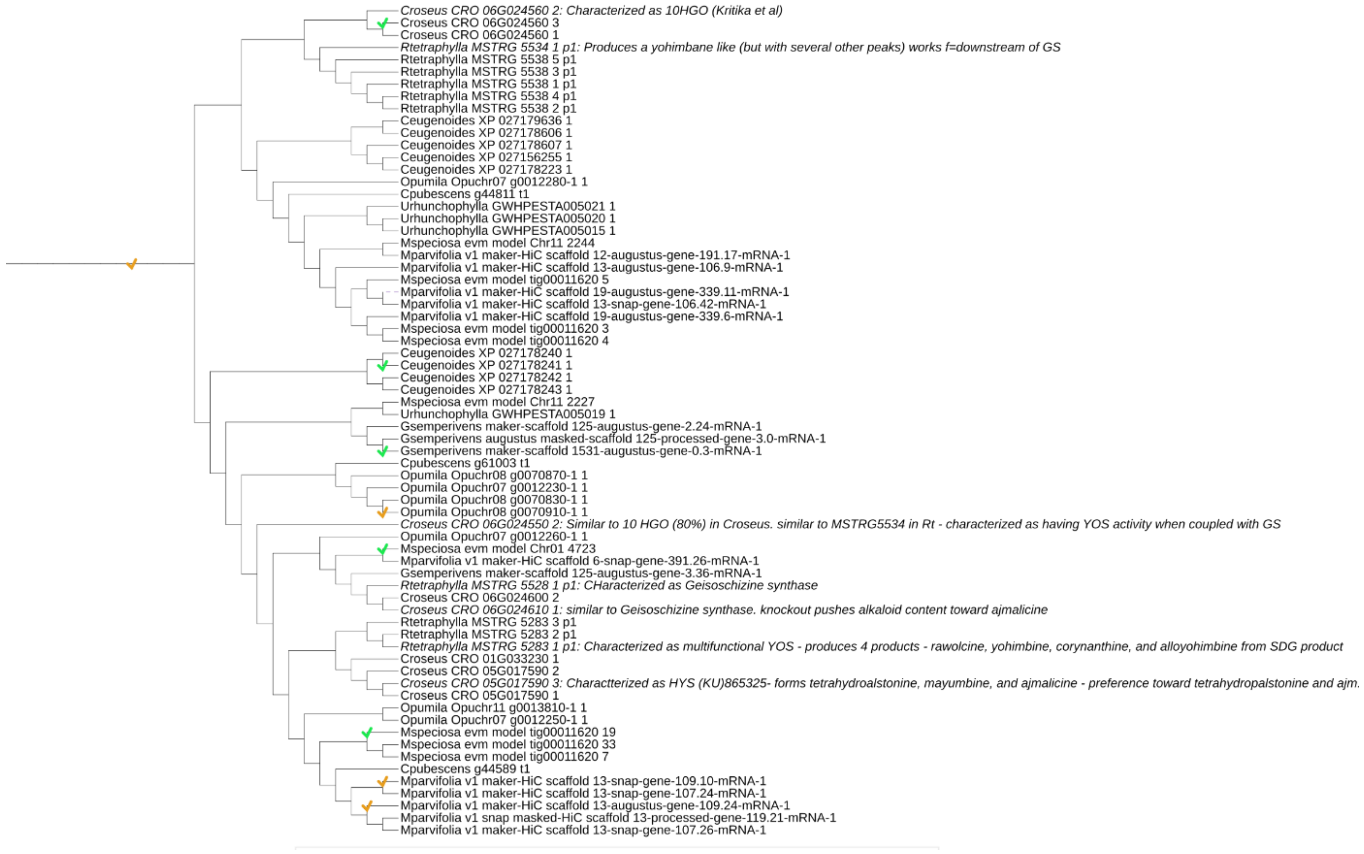

Supplementary Figure 13: Cladogram of *MDR* gene family, and ASBERL results indicating diversifying selection on branches of Gentianales specific clade. Related to main Fig. 3 and 4.

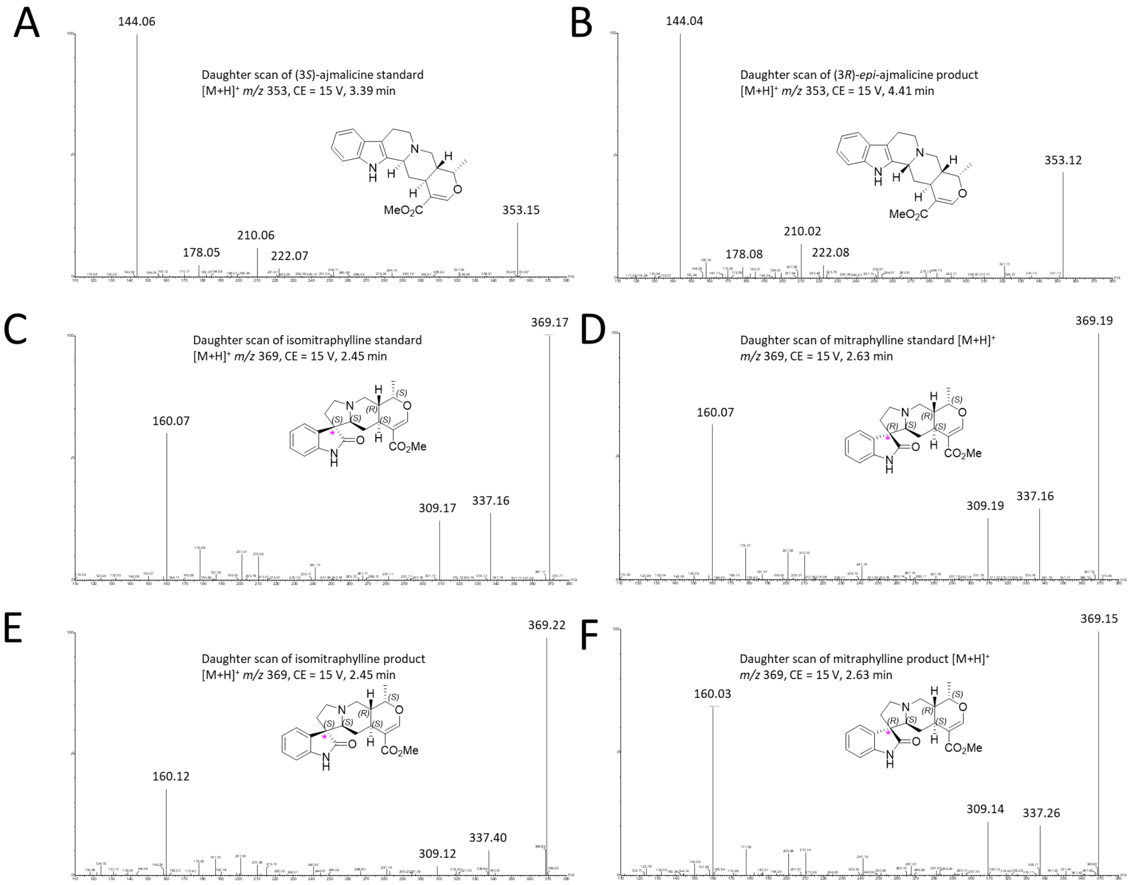

Supplementary Figure S14: Daughter scans and MS/MS spectra of A) authentic (3*S*)-ajmalicine standard, *m/z* $[M+H]^+$ 353, retention time (RT) 3.39 min, B) (3*R*)-*epi*-ajmalicine enzymatic product *m/z* $[M+H]^+$ 353, RT 4.41 min, C) and D) isomitraphylline standard *m/z* $[M+H]^+$ 369, RT 2.45 min and mitraphylline standard *m/z* $[M+H]^+$ 369, RT 2.63 min, E) and F) isomitraphylline and mitraphylline enzymatic products, *m/z* $[M+H]^+$ 369, RT 2.45 and 2.63 min, respectively. The fragmentation of (3*R*)-*epi*-ajmalicine product was identical to the (3*S*)-ajmalicine standard but elute at a different retention time. The fragmentation and retention time of the isomitraphylline and mitraphylline enzymatic products were identical to the authentic standards. Related to main Fig. 6.

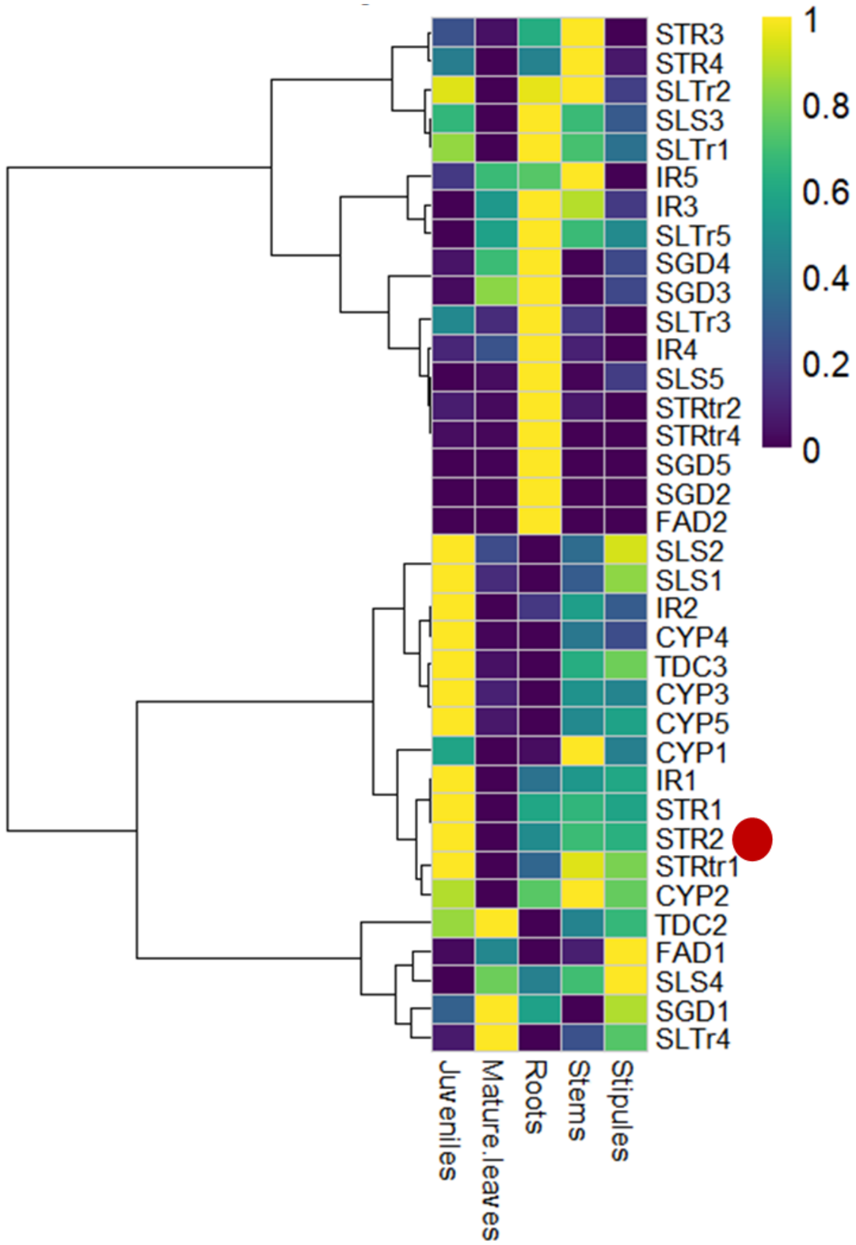

Supplementary Figure S15: Hierarchical clustering of MIA biosynthetic genes in *M. parvifolia* with the focus on the putative *MpSTR* (*STR2*). The highly correlated genes FAD1, IR1, CYP1 were selected for biochemical characterization. Among *CYP1*-*CYP5*, only *CYP1* belonged to the CYP71 family and had biochemical activity. The other *CYP2*-*CYP5* showed no activity among the tested *in vivo* combined assays with *FAD1* and *IR1* in *N. benthamiana*. Related to main Fig. 5 and 6. CYP: cytochrome P450, FAD: FAD-dependent oxidase, STR: strictosidine synthase, SGD: strictosidine β-D-glucosidase, TDC: tryptophan decarboxylase, SLS: secologanin synthase SLTr: secologanin transporter, IR: isoflavone reductase.

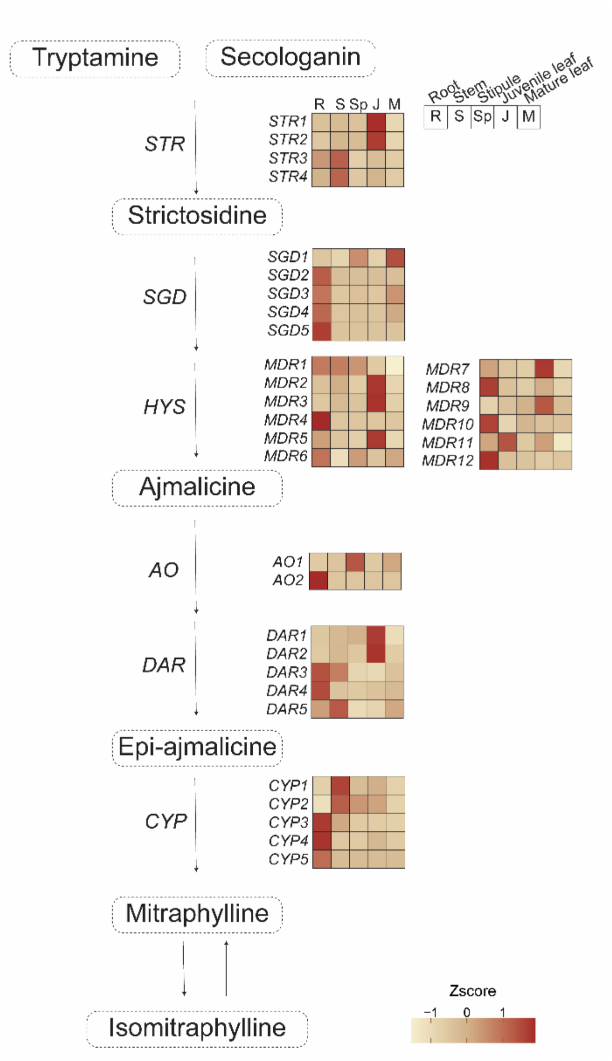

Supplementary Figure S16: Expression patterns of MIA biosynthetic pathway in *M. parvifolia* depicted as deviation from average expression (Z-score).*STR: strictosidine synthase*, *SGD: strictosidine β-D-glucosidase*, *HYS: Heteroyohimbine synthase*, *AO: Ajmalicine oxidase, DAR : Dehydroajmalicine reductase, CYP: Cytochrome P450.* Related to main Fig. 5 and 6.

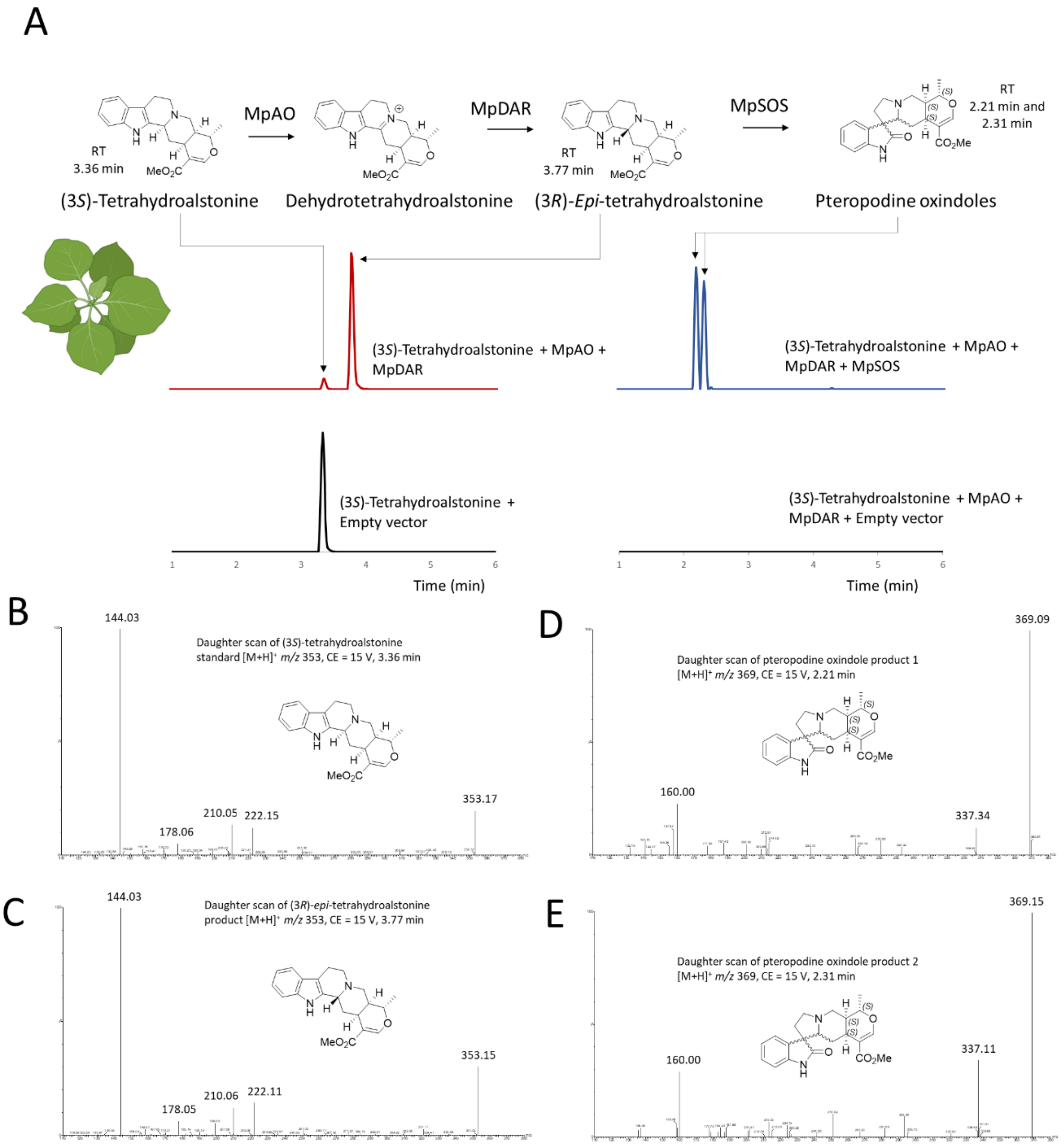

Supplementary Figure S17: A) Biochemical activity of *Mp*AO, *Mp*DAR and *Mp*SOS in *N. benthamiana* with (3*S*)-tetrahydroalstonine producing the pteropodine oxindole alkaloids. Extracted ion chromatograms (EIC) for *m/z* $[M+H]^+$ 353 showing that M*p*AO, *Mp*DAR can convert (3*S*)-tetrahydroalstonine (RT 3.36 min) to its (3*R*) isomer (RT 3.77 min), which was then converted to pteropodine oxindole and its isomer (RT 2.21 min and 2.31 min) by *Mp*SOS (EIC for *m/z* $[M+H]^+$ 369). Daughter scans and MS/MS spectra of B) (3*S*)-tetrahydroalstonine standard *m/z* $[M+H]^+$ 353, RT 3.31 min, C) (3*R*)-*epi*-tetrahydroalstonine enzymatic product *m/z* $[M+H]^+$ 353, RT 3.77 min, D) and E) pteropodine oxindole products 1 and 2 *m/z* $[M+H]^+$ 369, RT 2.21 min and 2.31 min.

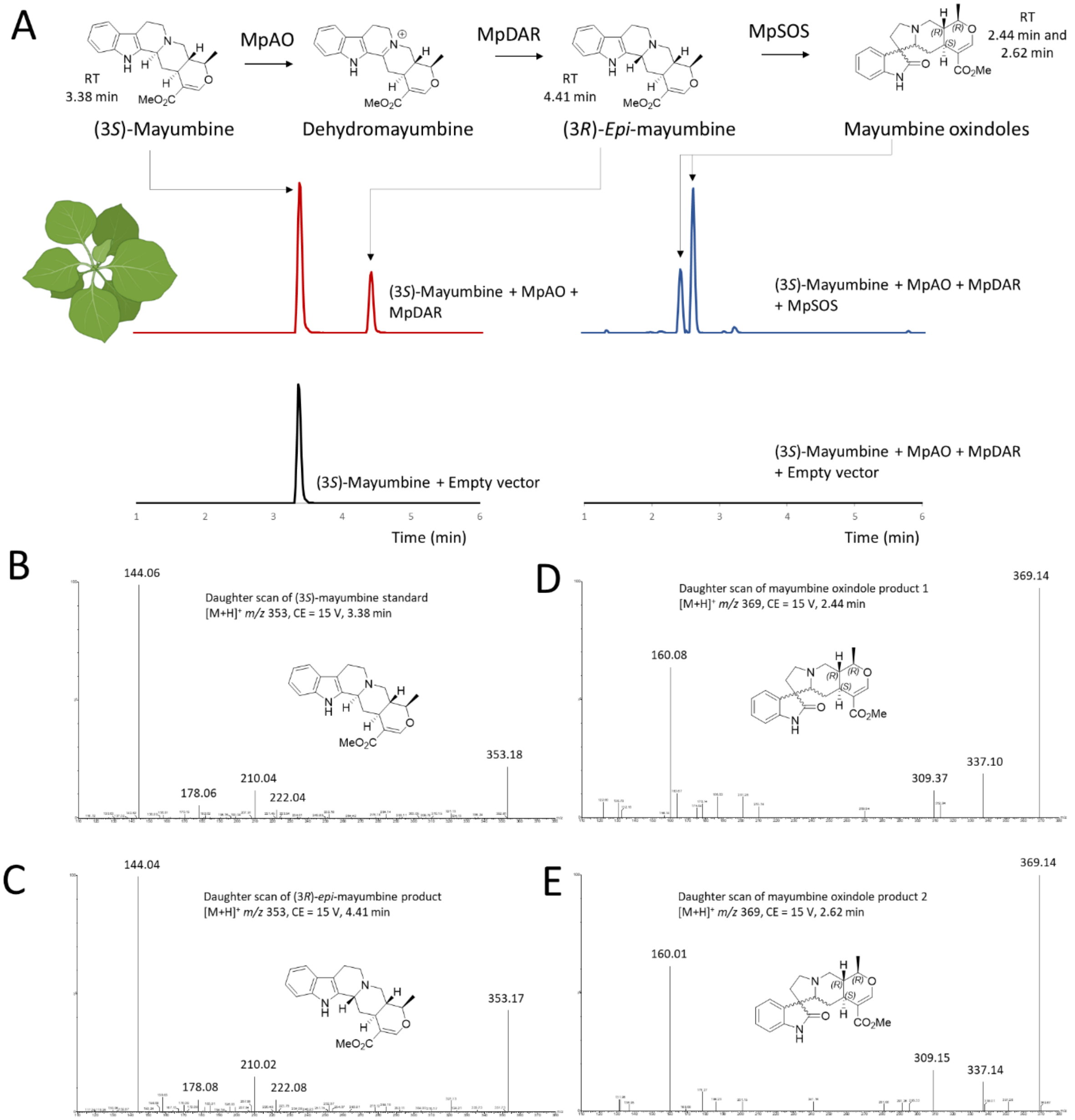

Supplementary Figure S18: A) Biochemical activity of *Mp*AO, *Mp*DAR and *Mp*SOS in *N. benthamiana* with (3*S*)-mayumbine producing the mayumbine oxindole alkaloids. Extracted ion chromatograms (EIC) for *m/z* $[M+H]^+$ 353 showing that M*p*AO, *Mp*DAR can convert (3*S*)-mayumbine (RT 3.38 min) to its (3*R*) isomer (RT 4.41 min), which was then converted to mayumbine oxindole and its isomer (RT 2.44 min and 2.62 min) by *Mp*SOS (EIC for *m/z* $[M+H]^+$ 369). Daughter scans and MS/MS spectra of B) (3*S*)-mayumbine standard *m/z* $[M+H]^+$ 353, RT 3.38 min, C) (3*R*)-*epi*-mayumbine enzymatic product *m/z* $[M+H]^+$ 353, RT 4.41 min, D) and E) mayumbine oxindole products 1 and 2 *m/z* $[M+H]^+$ 369, RT 2.44 min and 2.62 min.

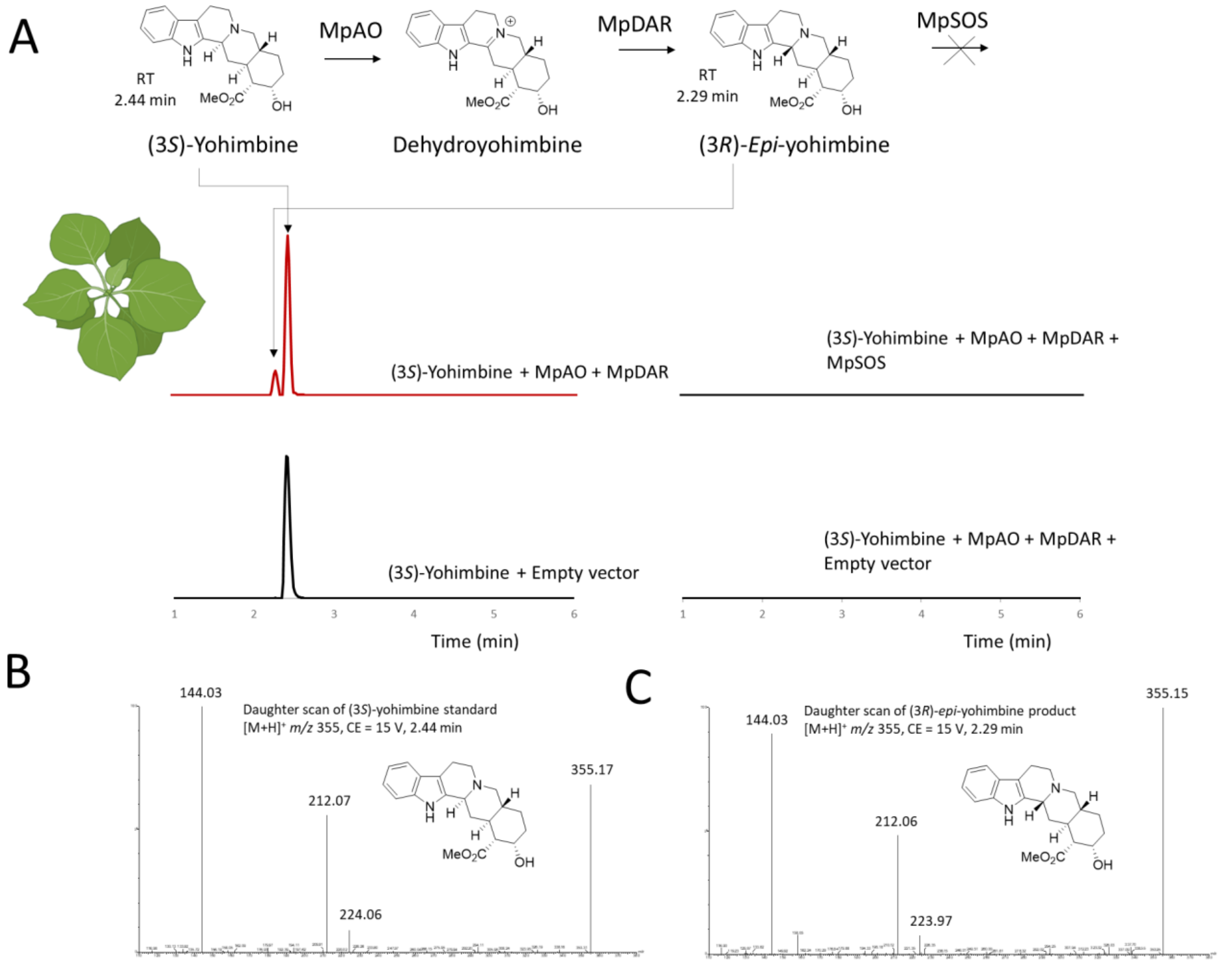

Supplementary Figure S19: A) Biochemical activity of *Mp*AO, *Mp*DAR, and *Mp*SOS in *N. benthamiana* with (3*S*)-yohimbine. Extracted ion chromatograms (EIC) for *m/z* $[M+H]^+$ 355 showing that *Mp*AO, *Mp*DAR can convert (3*S*)-yohimbine (RT 2.44 min) to its (3*R*) isomer (RT 2.29 min), this product was not accepted by *Mp*SOS, therefore no spirooxindole (EIC *m/z* $[M+H]^+$ *m/z* 371) were formed. Daughter scans and MS/MS spectra of B) (3*S*)-yohimbine standard *m/z* $[M+H]^+$ 355, RT 2.44 min, and C) (3*R*)-*epi*-yohimbine enzymatic product *m/z* $[M+H]^+$ 355, RT 2.29 min.

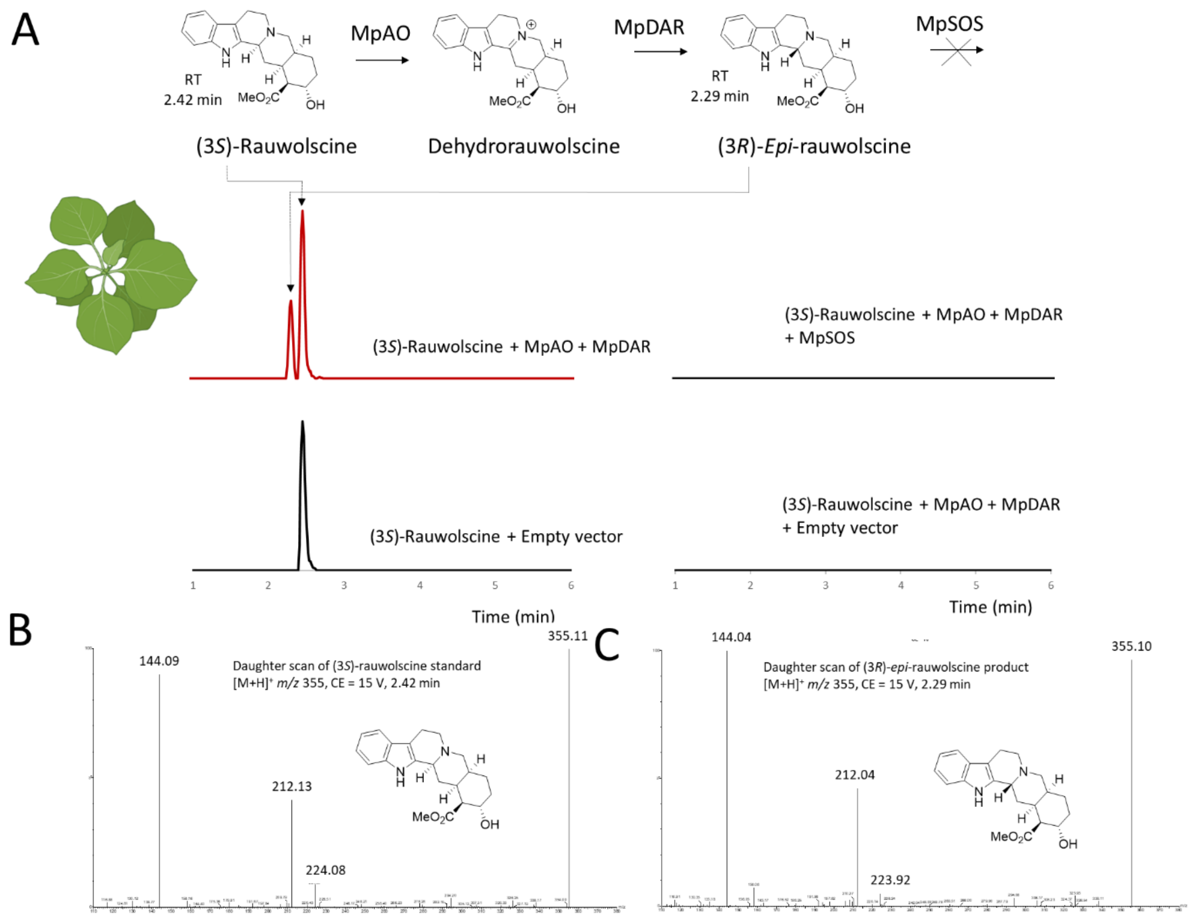

Supplementary Figure S20: A) Biochemical activity of *Mp*AO, *Mp*DAR, and *Mp*SOS in *N. benthamiana* with (3*S*)-rauwolscine. Extracted ion chromatograms (EIC) for *m/z* $[M+H]^+$ 355 showing that *Mp*AO, *Mp*DAR can convert (3*S*)-rauwolscine (RT 2.42 min) to its (3*R*) isomer (RT 2.29 min), this product was not accepted by *Mp*SOS, therefore no spirooxindole (EIC *m/z* $[M+H]^+$ *m/z* 371) were formed. Daughter scans and MS/MS spectra of B) (3*S*)-rauwolscine standard *m/z* $[M+H]^+$ 355, RT 2.42 min, and C) (3*R*)-*epi*- rauwolscine enzymatic product *m/z* $[M+H]^+$ 355, RT 2.29 min.

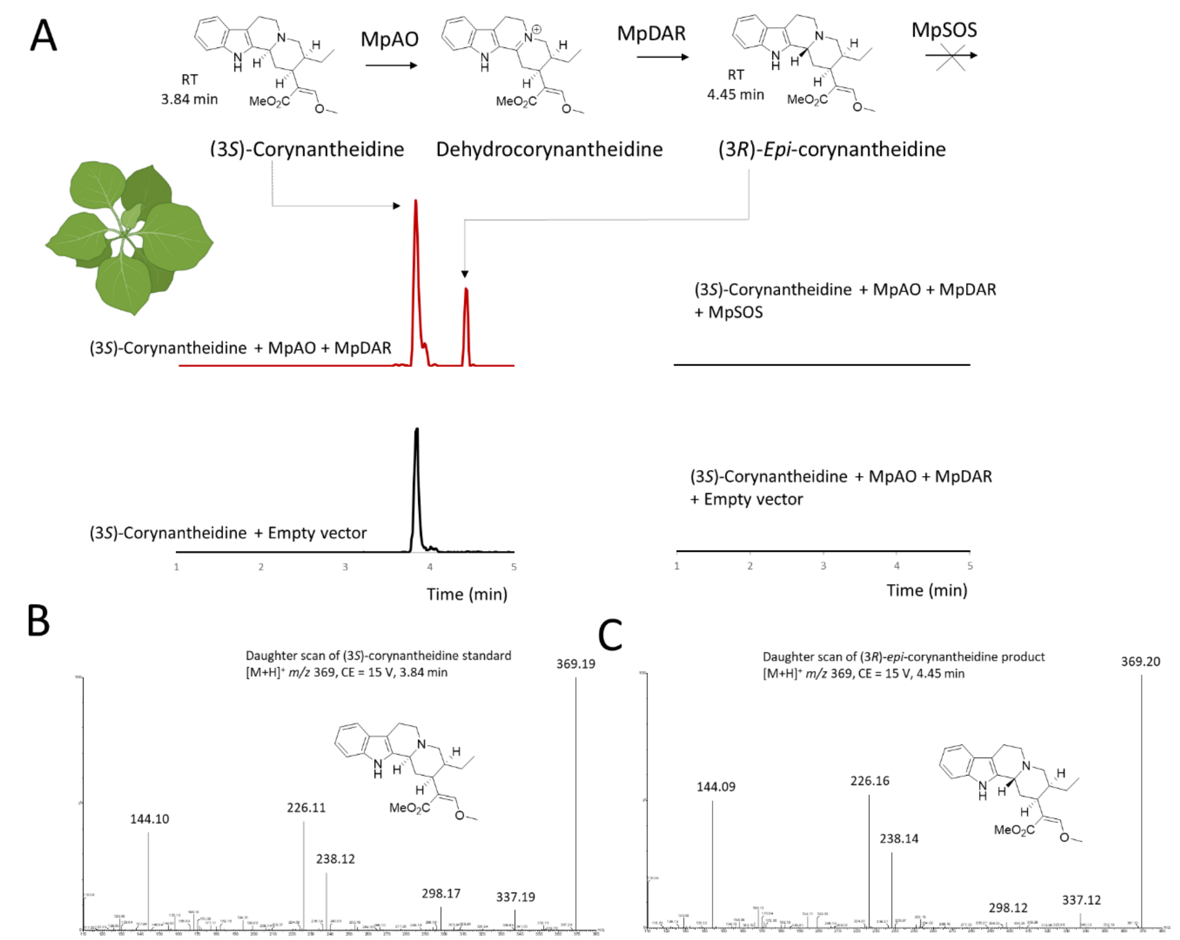

Supplementary Figure S21: A) Biochemical activity of *Mp*AO, *Mp*DAR, and *Mp*SOS in *N. benthamiana* with (3*S*)-corynantheidine. Extracted ion chromatograms (EIC) for *m/z* $[M+H]^+$ 369 showing that *Mp*AO, *Mp*DAR can convert (3*S*)-corynantheidine (RT 3.84 min) to its (3*R*) isomer (RT 4.45 min), this product was not accepted by *Mp*SOS, therefore no spirooxindole (EIC *m/z* $[M+H]^+$ *m/z* 385) were formed. Daughter scans and MS/MS spectra of B) (3*S*)-corynantheidine standard *m/z* $[M+H]^+$ 369, RT 3.84 min, and C) (3*R*)-*epi*-corynantheidine enzymatic product *m/z* $[M+H]^+$ 369, RT 4.45 min.

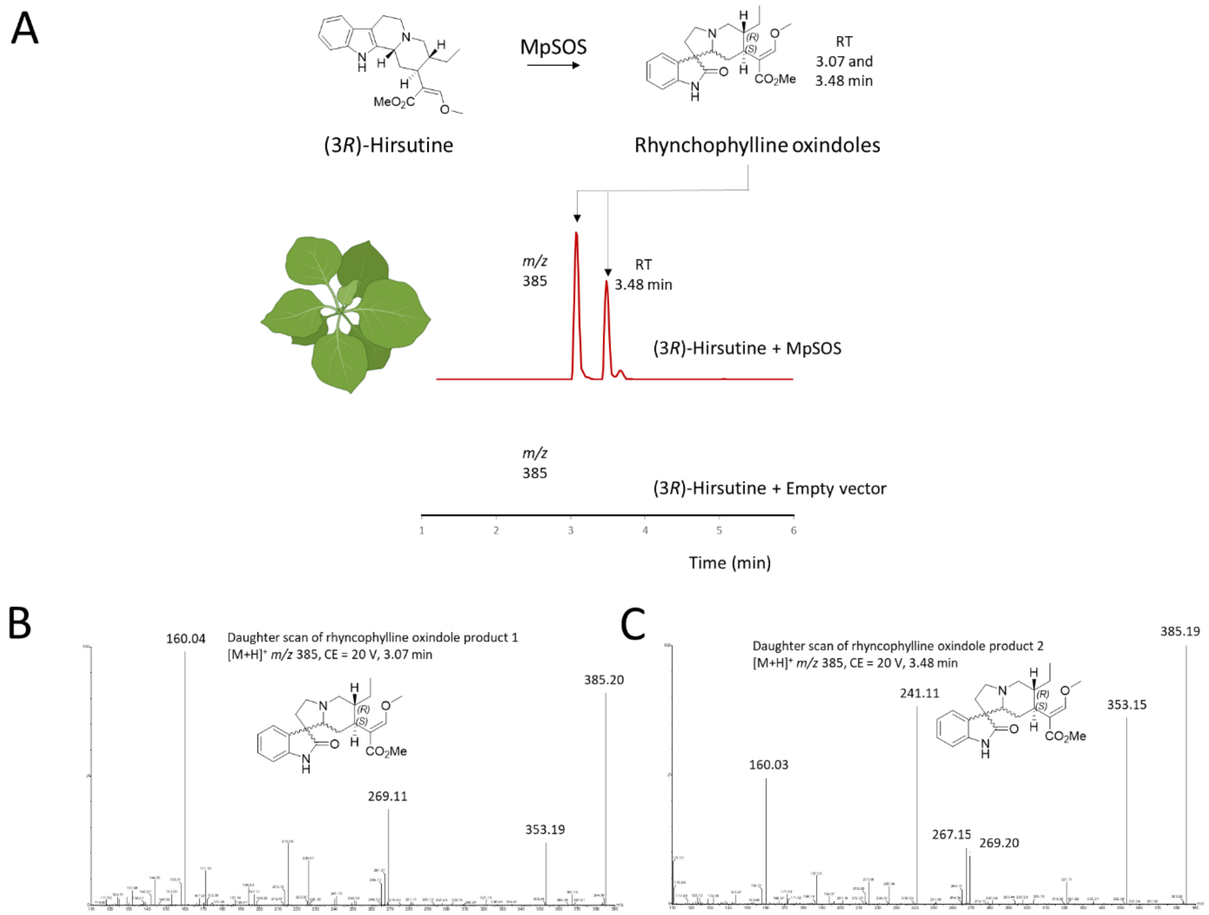

Supplementary Figure S22: A) Biochemical activity *MpSOS* in *N. benthamiana* with substrate (3*R*)-hirsutine producing the rhynchophylline-type spirooxindoles (EIC *m/z* $[M+H]^+$ 385). Daughter scans and MS/MS spectra of B) rhynchophylline oxindole product 1 *m/z* $[M+H]^+$ 385, RT 3.07 min, and C) rhynchophylline oxindole product 2 *m/z* $[M+H]^+$ 385, RT 3.48 min.

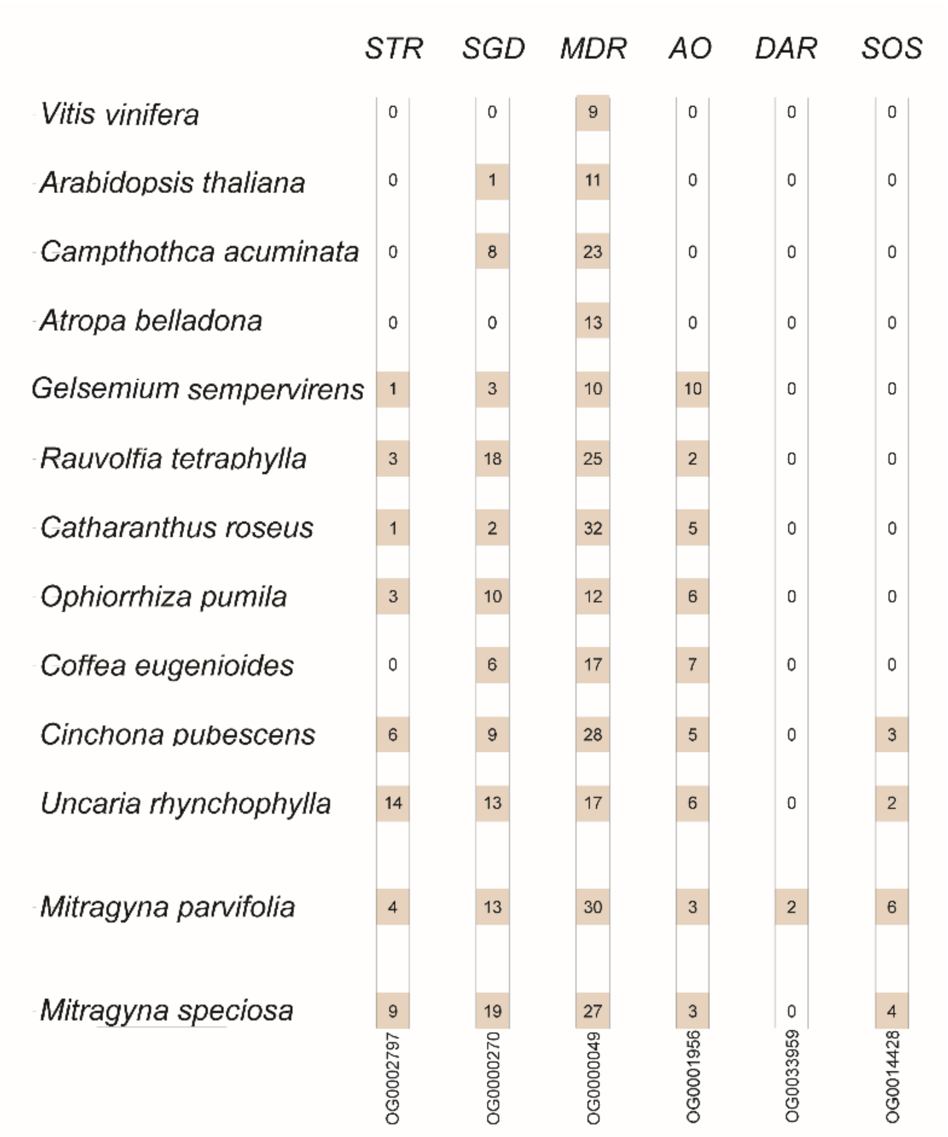

Supplementary Figure S23: Distribution of MIA biosynthetic enzyme gene families in different species. MpDAR is specific for rich mitraphylline producing species *M. parvifolia. STR: strictosidine synthase*, *SGD: strictosidine β-D-glucosidase*, *MDR: medium chain reductase* , *AO: Ajmalicine oxidase, DAR : Dehydroajmalicine reductase, SOS: spirooxindole synthase.*

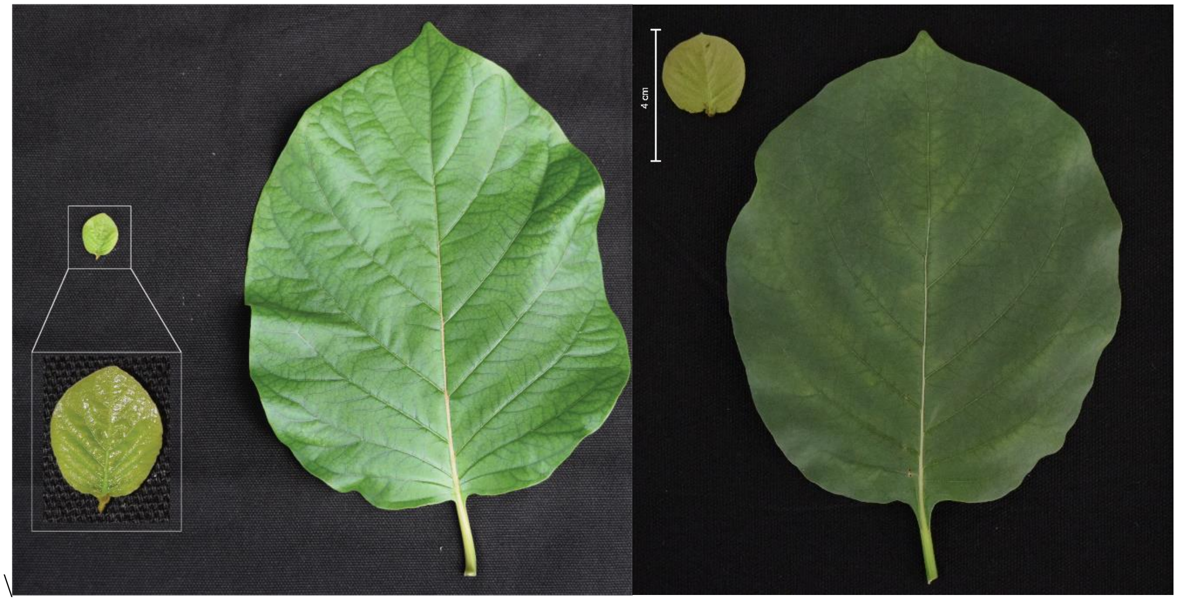

\

Supplementary Figure S24: Examples of *Mitragyna parvifolia* young (left) and mature (right) leaves.
